# Ageing promotes metastasis via activation of the integrated stress response

**DOI:** 10.1038/s41586-026-10216-0

**Published:** 2026-03-11

**Authors:** Angana A. H. Patel, Jozefina J. Dzanan, Kevin X. Ali, Ella A. Eklund, Samantha W. Alvarez, Dorota Raj, Martin Dankis, Ilayda Altinönder, Maria Schwarz, Kristell Le Gal, Emre Bedel, Ahmed Ezat El Zowalaty, Emma Jonasson, Heba Albatrok, Nadia Gul, Jozef P. Bossowski, Ray Pillai, Patrick Micke, Johan Botling, Levent M. Akyürek, Davide Angeletti, Sama I. Sayin, Anetta Härtlova, Thales Papagiannakopoulos, Roger Olofsson Bagge, Anders Ståhlberg, Andreas Hallqvist, Clotilde Wiel, Volkan I. Sayin

**Affiliations:** 1 Sahlgrenska Center for Cancer Research, Department of Surgery, Institute of Clinical Sciences, University of Gothenburg, Gothenburg, Sweden; 2 Wallenberg Centre for Molecular and Translational Medicine, University of Gothenburg, Gothenburg, Sweden; 3 Department of Oncology, Sahlgrenska University Hospital, Gothenburg, Sweden; 4 Department of Nutritional Physiology, Institute of Nutritional Sciences, Friedrich Schiller University Jena, Jena, Germany; 5 Sahlgrenska Center for Cancer Research, Department of Laboratory Medicine, Institute of Biomedicine, University of Gothenburg, Gothenburg, Sweden; 6 Department of Pathology, New York University Grossman School of Medicine, New York, NY USA; 7 Perlmutter Cancer Center, New York University Grossman School of Medicine, New York, NY USA; 8 Department of Immunology, Genetics, and Pathology, Uppsala University, Uppsala, Sweden; 9 Department of Laboratory Medicine, Institute of Biomedicine, University of Gothenburg, Gothenburg, Sweden; 10 Department of Clinical Pathology, Sahlgrenska University Hospital, Gothenburg, Sweden; 11 Department of Microbiology and Immunology, Institute of Biomedicine, Sahlgrenska Academy/Faculty of Science, University of Gothenburg, Gothenburg, Sweden; 12 Institute of Medical Microbiology and Hygiene, Faculty of Medicine, Medical Center - University of Freiburg, Freiburg, Germany; 13 Department of Clinical Genetics and Genomics, Sahlgrenska University Hospital, Region Västra Götaland, Gothenburg, Sweden; 14 Science for Life Laboratory, Institute of Biomedicine, University of Gothenburg, Gothenburg, Sweden

**Keywords:** Cancer metabolism, Non-small-cell lung cancer, Metastasis, Mechanisms of disease, Ageing

## Abstract

Lung cancer predominantly affects older individuals, yet how physiological ageing influences tumour evolution remains poorly understood^1^. Here we show that ageing reprograms the evolutionary trajectory of *KRAS*-driven lung adenocarcinoma, limiting primary tumour growth while promoting metastatic dissemination through epigenetic activation of the integrated stress response (ISR). The ISR effector ATF4 drives epithelial and metabolic plasticity, conferring metastatic competence. Mechanistically, aged tumour cells show increased sensitivity to the PERK–eIF2α arm of the unfolded protein response, sustaining persistent ATF4 signalling. Targeting ISR–ATF4 genetically or pharmacologically abolishes these adaptations and limits dissemination, whereas ATF4 overexpression alone is sufficient to induce metastasis. The ageing–ATF4 axis imposes a dependency on glutamine metabolism, revealing a therapeutically actionable vulnerability. Clinical analyses confirm that ATF4 is enriched in aged tumours and correlates with poor survival and advanced-stage disease. Collectively, these results define epigenetic ISR–ATF4 activation as a causal driver of lineage plasticity and metastasis in aged tumours, revealing a therapeutic opportunity in older patients with lung adenocarcinoma, the most common yet understudied subset of lung cancer.

## Abstract (web summary)

Ageing reprograms the evolutionary trajectory of *KRAS*-driven lung adenocarcinoma, limiting primary tumour growth while promoting metastatic dissemination through epigenetic activation of the integrated stress response, and a therapeutic opportunity in older patients is revealed.

## Main

Ageing and cancer share core hallmarks, including loss of proteostasis, epigenetic remodelling and altered nutrient sensing^2^, and epidemiological data link increasing age to higher cancer incidence and mortality. Despite this association, the mechanisms by which ageing shapes cancer progression remain poorly defined.

Metastasis accounts for roughly 90% of cancer-related deaths^3,4^, yet how ageing affects metastatic competence is unknown. Lung cancer, the leading cause of cancer-related deaths worldwide^1^, primarily affects older individuals and is often diagnosed at metastatic stage, with a median age of 70 years and peak incidence between 65 years and 75 years^5^. However, in vivo mechanistic studies of lung cancer almost exclusively use young mice^6^, overlooking the physiological context of ageing that defines most patients with lung cancer. This age disconnect between experimental models and patients may explain why many cancer therapies that perform well preclinically, such as with glutaminase (GLS) inhibitors (GLSi), have failed to translate their efficacy in clinical trials^7,8^.

Age-associated changes in humans are largely conserved in mice, making them an excellent model for studying mammalian ageing^9^. In addition, genetically engineered mouse models (GEMMs) such as the *Kras*^*LSL-G12D/+*^*;Trp*53^*flox/flox*^ (*KP*) model faithfully recapitulate human lung tumour development within an intact microenvironment, providing a physiologically relevant system to study tumour progression and dissemination^10^. Emerging evidence indicates that age-related alterations in the tumour microenvironment can drive metastatic progression^11,12^, but how physiological ageing intrinsically reprograms lung cancer cells to affect disease trajectory remains unknown. Here we use young and old *KP* mice to mechanistically define the impact of physiological ageing on lung cancer progression and metastasis.

## Ageing alters lung cancer progression

**Fig. 1:**
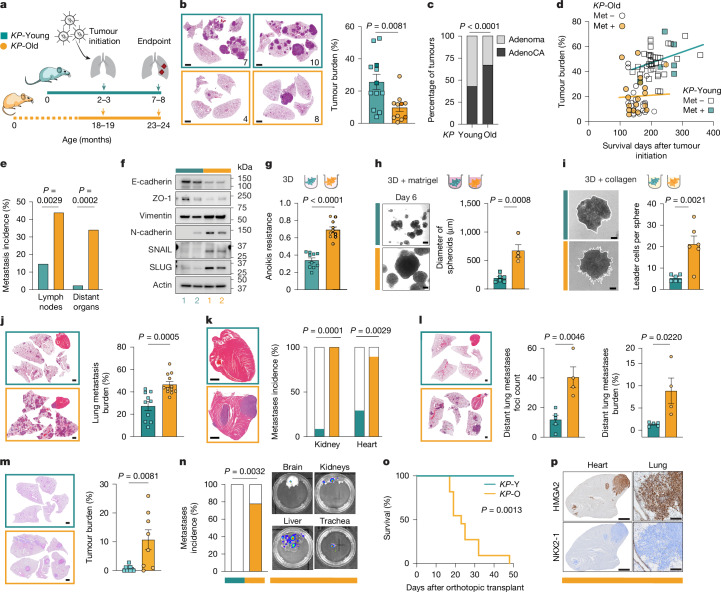
Ageing favours metastasis over primary lung tumour growth. **a**, Experimental timeline in *KP*-Young and *KP*-Old mice. **b**, Representative haematoxylin and eosin (H&E)-stained lung sections and tumour burden at 21 weeks after tumour initiation (*KP*-Young, *n* = 12; *KP*-Old, *n* = 11). **c**, Proportion of adenomas and adenocarcinomas (AdenoCA) in *KP*-Young and *KP*-Old tumours (*n* = 391 and *n* = 97). **d**, Correlation of primary lung tumour burden with days after Lenti-Cre in *KP*-Young (*n* = 42, squares, *P* = 0.0531) and *KP*-Old (*n* = 41, circles, *P* = 0.7026). Mice with metastasis (Met) are indicated. **e**, Incidence of local lymph node and distant metastases in the cohort shown in **d**. **f**, Western blot analysis of EMT markers in *KP*-Y and *KP*-O (*n* = 2). **g**, Anoikis resistance of *KP*-Y and *KP*-O (*n* = 10). **h**, Representative spheroid images and quantification of spheroid diameter of *KP*-Y (*n* = 6) and *KP*-O (*n* = 4). **i**, Representative images and quantification of leader cells per spheroid formed by *KP*-Y (*n* = 6) and *KP*-O (*n* = 6). **j**,**k**, H&E-stained lung sections and lung metastasis burden (**j**), and representative H&E-stained heart sections and metastasis incidence in the kidney and heart (**k**) following intravenous injection of *KP*-Y (*n* = 10) and *KP*-O (*n* = 12) in mice. **l**, Distant lung metastasis after subcutaneous injection of *KP*-Y (*n* = 5) and *KP*-O (*n* = 4). Left, representative H&E-stained lung and heart sections. Middle, lung foci number. Right, lung metastasis burden. **m**, Representative H&E-stained lungs and tumour burden 28 days after orthotopic transplantation of *KP*-Y (*n* = 9) and *KP*-O (*n* = 8). **n**, Distant metastases following orthotopic transplantation of *KP*-Y (*n* = 7) and *KP*-O (*n* = 9). Right, representative ex vivo bioluminescence images of distant organ metastases. **o**, Survival of mice orthotopically transplanted with *KP*-Y (*n* = 4) and *KP*-O (*n* = 11). **p**, Representative HMGA2 and NKX2-1 IHC staining of heart (left) and lung (right) metastases formed by *KP*-O. Data are mean ± s.e.m. Two-tailed unpaired *t*-test (**b**,**g**,**h**–**j**,**l**,**m**); two-sided Chi-square test (**c**,**e**,**k**), Simple linear regression (**d**), two-sided Fisher’s exact test (**n**) and log-rank test (**o**). Scale bars, 2 mm (**b**); 200 μm (**h**,**i**,**p** (right)); 1 mm (**j**–**m**,**p** (left)). Source data

**Extended Data Fig. 1:**
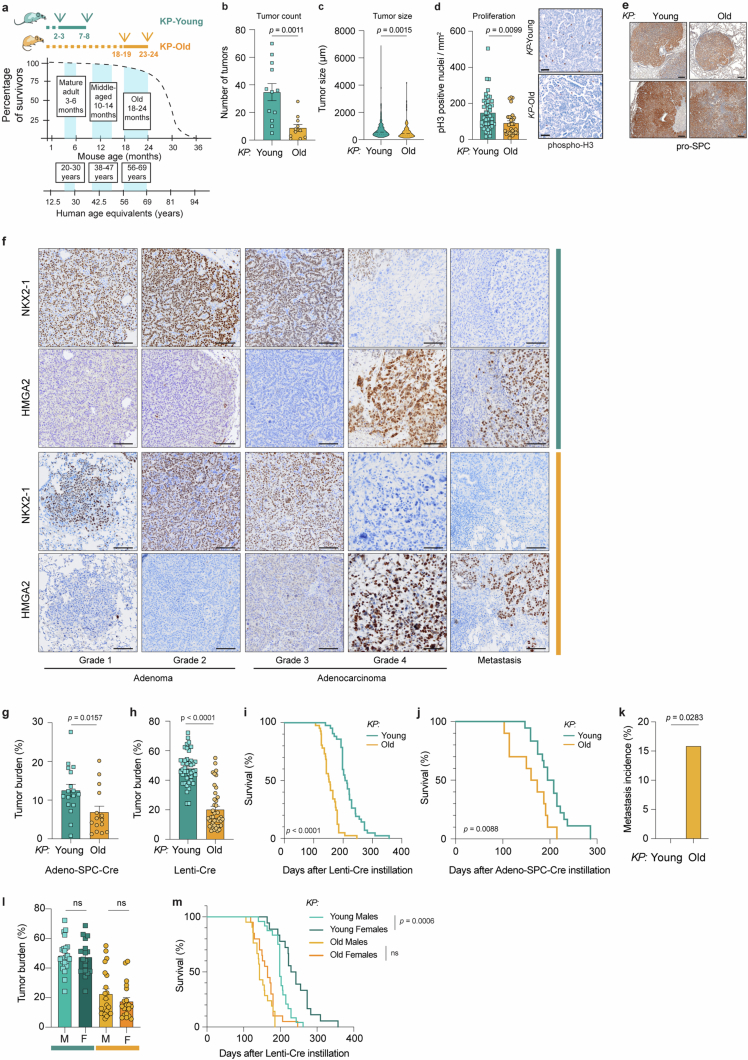
Ageing impacts KRAS-driven lung tumor development in mice and their survival. **a**, Schematic showing mouse-human age equivalences (adapted from^78^). Timeline of the in vivo experimental setup is shown on top, indicating tumor initiation and endpoint in *KP*-Young and *KP*-Old mice. **b-c**, Tumor number (*n* = 12 and 11; **b**) and tumor size (*n* = 391 and 97; **c**) in *KP*-Young and *KP*-Old mice at 21 weeks after instillation with Lenti-Cre. **d**, Quantification of phosphorylated histone H3 (pH3)-positive nuclei per squared millimeter in lung tumors from *KP*-Young (*n* = 43 tumors, from 6 mice) and *KP*-Old (*n* = 27 tumors, from 6 mice) mice at 21 weeks after instillation with Lenti-Cre. Representative pH3 immunohistochemistry (IHC) staining images are shown (Scale bars, 50 µm). **e**, Representative IHC for alveolar type II (AT2) cell lineage marker Surfactant Protein-C (pro-SPC) in *KP*-Young and *KP*-Old. Scale bars, 200 µm. **f**, Representative IHC for NKX2-1 and HMGA2 from *KP* tumors in and lymph node metastases in *KP*-Young (top) and *KP*-Old (bottom) (Scale bars, 100 µm). **g**, Lung tumor burden 17 weeks after tumor initiation with Adeno-SPC-Cre in *KP* -Young (*n* = 17) and *KP*-Old (*n* = 15) mice. **h-i**, Tumor burden (**h**) and survival (**i**) of *KP*-Young (*n* = 42) and *KP*-Old (*n* = 41) mice after tumor initiation with Lenti-Cre, from the cohort and data shown in Fig. 1d). Median survival is 210 and 152 days for *KP-*Young and *KP*-Old respectively. **j**, Survival of *KP*-Young and *KP*-Old mice after tumor initiation with Adeno-SPC-Cre virus. Median survival is 202 and 167 days for *KP* -Young (*n* = 18) and *KP* -Old (*n* = 10) respectively. **k**, Incidence of local lymph nodes metastases in *KP-*Young and *KP*-Old. Data shown are from time points experiments **l-m**, Tumor burden *KP-*Young (*n* = 24, males and *n* = 18, females) and *KP*-Old (*n* = 21, males and *n* = 20, females) (**l**) and survival (**m**) of mice as in (**h,i**), males and females separated. ns: not significant. Data are mean values ± s.e.m, and as median for (**c**). Two-tailed unpaired t-test (**b,d,e,g**); two-sided Kolmogorov-Smirnov test (**c**) Log-rank (Mantel-Cox) test (**i,j,m**), two-sided Chi-square test (**k**), and one-way ANOVA with Sidak’s multiple comparisons test (**l**). Schematic in **a** adapted with permission from ref. ^78^, Elsevier. Source data

To investigate the impact of ageing on lung tumorigenesis, we simultaneously induced lung tumours in *KP* mice aged 2–3 months (*KP*-Young) and 18–19 months (*KP*-Old) by means of intratracheal instillation of Cre-encoding viral particles (Fig. 1a). These age groups represent early adulthood, which is the typical age used in lung cancer studies^10^, and an older age at which molecular ageing phenotypes become relevant^1,13,14^. The age of the old group following a 5–6 month experiment corresponds to the median age at diagnosis for non-small cell lung cancer (NSCLC) at 70 years^1^, thus accurately modelling the most common demographic of human NSCLC (Extended Data Fig. 1a) and overcoming limitations of conventional GEMMs that omit host age^14^. We observed a 2.5-fold decrease in primary lung tumour burden in *KP*-Old mice compared with *KP*-Young mice (Fig. 1b). Histological and immunohistochemical (IHC) analyses revealed fewer, smaller and less proliferative tumours in *KP*-Old mice. In addition, all tumours from *KP*-Old and *KP*-Young mice were positive for the alveolar type 2 (AT2) lineage marker pro-SPC (Extended Data Fig. 1b–e). We observed a higher proportion of adenocarcinomas following the canonical dedifferentiation trajectory^15^ in *KP*-Old mice compared with *KP*-Young mice (Fig. 1c and Extended Data Fig. 1f), indicating that malignant disease progression is accelerated in *KP*-Old mice despite lower lung tumour burden. Reduced tumour burden in *KP*-Old mice compared with *KP*-Young was confirmed using an AT2-specific Adeno-SPC-Cre, only recombining *KP* in AT2 cells^16^ (Extended Data Fig. 1g).

Longitudinal analysis revealed that primary lung tumour burden increased linearly over time in *KP*-Young mice (140–357 days), whereas *KP*-Old mice maintained low tumour burden across the analysed time points (106–247 days), yet showed early incidence of metastasis and decreased survival (Fig. 1d and Extended Data Fig. 1h–j). Indeed, necropsy revealed a markedly higher incidence of metastases in local lymph nodes and distant organs in *KP-*Old mice versus *KP*-Young mice (Fig. 1e and Extended Data Fig. 1k). Although no difference in tumour burden and survival was observed between the sexes in old mice, young males showed significantly shorter survival compared with young females (Extended Data Fig. 1l,m).

**Extended Data Fig. 2:**
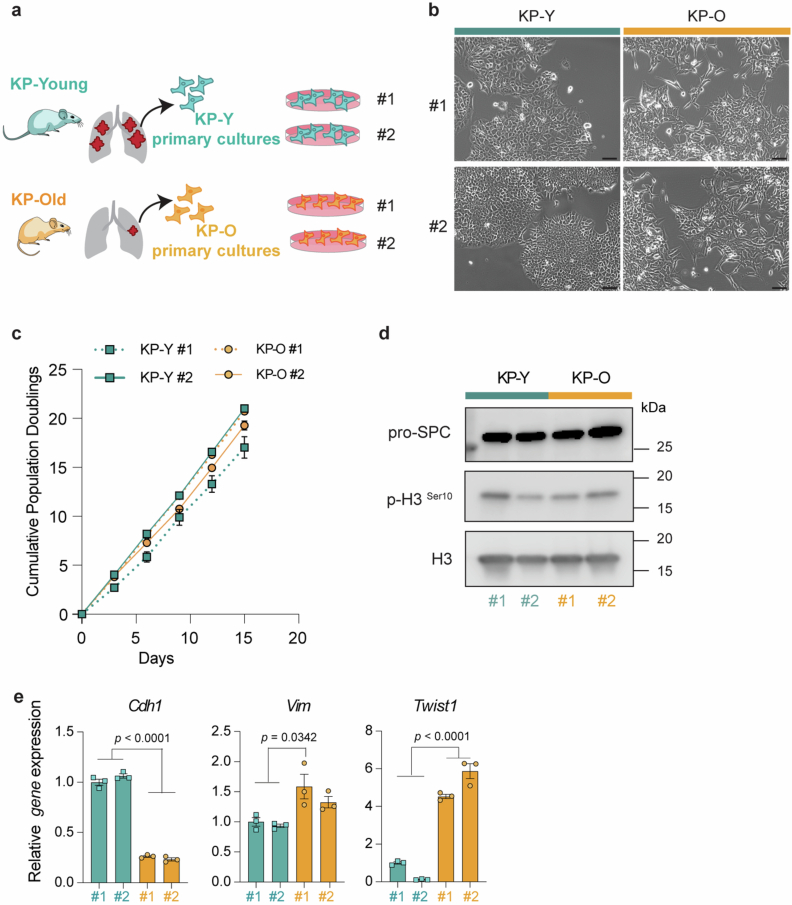
Establishment of primary tumor cultures from *KP*-Young and *KP*-Old mice. **a**, Lung tumors from *KP*-Young and *KP*-Old mice were dissociated to establish primary tumor cultures (*KP*-Y, teal and *KP*-O, gold). Two independent primary cultures were generated per group. **b**, Representative brightfield images of primary cultures *KP*-Y and *KP*-O (*n* = 2, scale bars, 200 µm). **c**, Cumulative population doublings of *KP*-Y and *KP*-O over 15 days (*n* = 4). **d**, Western blot of proliferation marker p-HISTONE3 (p-H3) and pneumocyte type II (AT2 cells) lineage marker Surfactant Protein-C (pro-SPC) in *KP*-Y and *KP*-O. **e**, Relative mRNA expression of EMT markers *Cdh1* (E-CADHERIN), *Vim* (VIMENTIN) and *Twist1* (TWIST) in *KP*-Y and *KP*-O (*n* = 3). Data are mean values ± s.e.m. (**c,e**). Ordinary one-way ANOVA with Tukey’s multiple comparisons (**e**).

**Extended Data Fig. 3:**
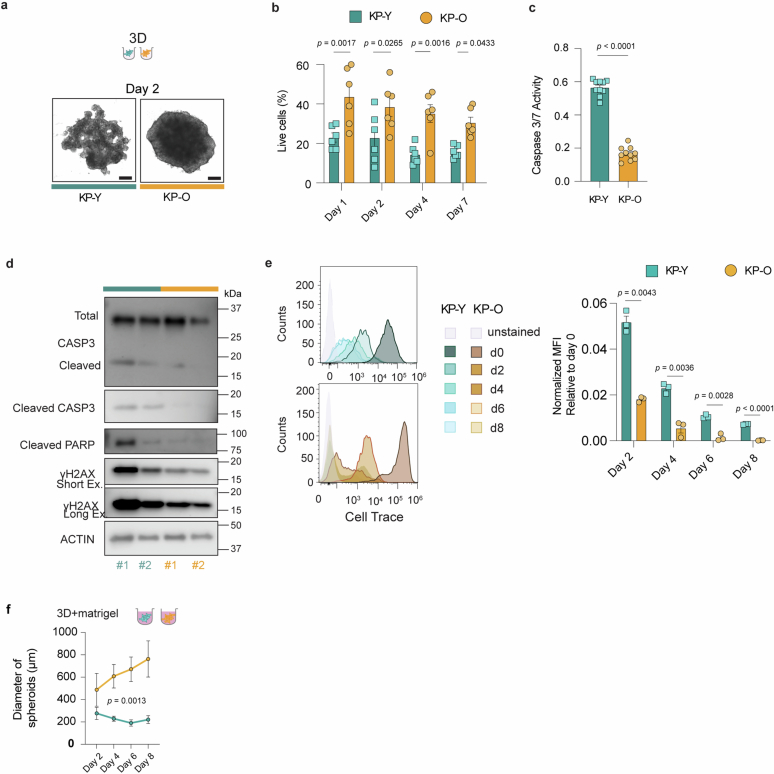
*KP*-O primary cultures are resistant to anoikis. **a**, Schematic and representative images of *KP*-Y and *KP*-O primary cultures seeded in ultra-low attachment plates (ULA). Scale bars, 200 µm. **b**, Viability (measured as trypan blue exclusion) of *KP*-Y and *KP*-O grown in ULA plates for 1, 2, 4 and 7 days (*n* = 6). Viability counts shown as percentage of live *KP*-Y and *KP*-O. **c**, Caspase 3/7 activity of *KP*-Y and *KP*-O grown in ULA for 48 h (*n* = 10). **d**, Western blot analysis of apoptosis and DNA damage markers of *KP*-Y and *KP*-O grown in ULA for 48 h. short ex: short exposure; long ex: long exposure. **e**, Cell Trace proliferation of *KP*-Y and *KP*-O (*n* = 3) grown in ULA plates. Left, representative curves obtained from the CellTrace at day 0, day 2, day 4, day 6, day 8. Right, quantification of Median Fluorescence Intensity (MFI), relative to day 0. **f**, Diameter of spheroids formed by *KP*-Y (*n* = 6) and *KP*-O (*n* = 4) measured on day 2, day 4, day 6 (shown in Fig. 1h) and day 8 after seeding in ULA plates with Matrigel. Data are mean values ± s.e.m. Two-way ANOVA (**b,f**); unpaired t-test (**c**) and two-sided multiple unpaired t-test (**e**).

To investigate why smaller and slower growing primary lung tumours from *KP*-Old mice progressed faster to metastatic lung adenocarcinomas, we next established primary tumour cultures (*n* = 2 + 2) from *KP*-Young and *KP*-Old mice (*KP*-Y and *KP*-O cultures respectively, Extended Data Fig. 2a,b). *KP*-Y and *KP*-O cultures showed comparable proliferation rates (Extended Data Fig. 2c,d) and similar levels of pro-SPC (Extended Data Fig. 2d), consistent with an AT2 origin. However, expression of epithelial–mesenchymal transition (EMT) markers were increased in *KP*-O cultures (Fig. 1f and Extended Data Fig. 2e), in line with a more metastatic profile. We next examined important features for metastatic dissemination, including growth in three-dimensional (3D) conditions and resistance to anoikis. *KP*-O cultures showed increased cell viability (Fig. 1g and Extended Data Fig. 3a,b), reduced levels of caspase 3/7 activity (Extended Data Fig. 3c,d) and augmented growth over a period of 8 days (Extended Data Fig. 3e). To model extracellular matrix–tumour cell interactions, *KP*-O and *KP*-Y cultures were embedded in Matrigel or collagen. *KP*-O cultures formed larger spheroids in Matrigel (Fig. 1h and Extended Data Fig. 3f), and they formed more invasive structures in collagen (Fig. 1i) compared with *KP*-Y cultures. Altogether, these data indicate increased metastatic propensity in *KP*-O cultures compared with *KP*-Y cultures in vitro.

In vivo tail-vein injection of *KP*-O cultures resulted in increased lung metastasis burden and higher incidence of kidney and heart metastases compared with *KP*-Y cultures (Fig. 1j,k). Although underscoring the diverse tropism of *KP*-O cultures, these findings did not inform further about their ability to disseminate and colonize distant sites. We therefore grafted *KP*-O and *KP*-Y cultures subcutaneously in the flanks of mice, and quantified distant metastasis. Histological analysis revealed more distant lung metastasis foci and increased lung metastasis burden in *KP*-O cultures (Fig. 1l), consistent with their increased metastatic capabilities. To further evaluate their ability to seed, grow and disseminate from their tissue of origin, we orthotopically transplanted *KP*-Y and *KP*-O cultures into the lungs of mice. *KP*-O cultures showed a significantly higher engraftment efficiency, a greater capacity to colonize distant organs including liver, kidneys and brain (80% of mice versus none of the mice engrafted with *KP*-Y) and markedly reduced survival time of recipient mice (Fig. 1m–o). In line with autochthonous tumours in *KP*-Old mice, metastases from *KP*-O cultures showed loss of NKX2-1 and gain of HMGA2 (Fig. 1p). Altogether, our findings indicate that ageing limits primary lung tumour growth in favour of promoting malignant disease progression in metastatic lung adenocarcinoma.

## Ageing enriches EMT and UPR pathways

**Fig. 2:**
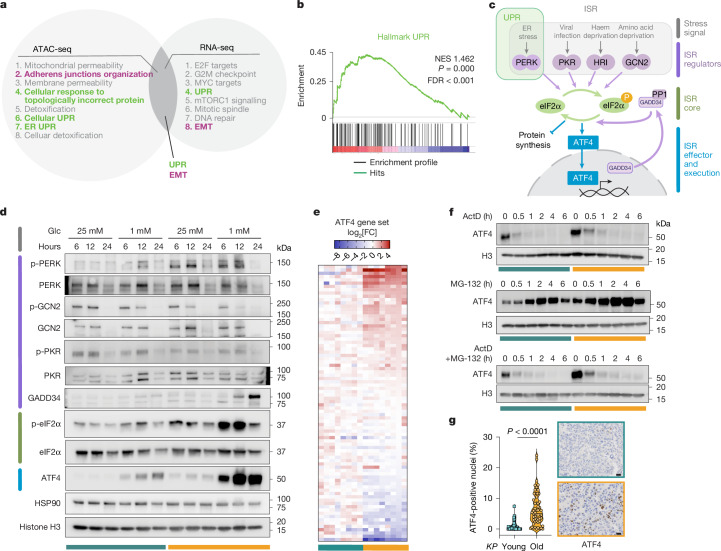
Sustained epigenetic induction of UPR–ISR–ATF4 in old tumours. **a**, Top enriched pathways identified by GREAT analysis of ATAC-seq and GSEA from RNA-seq in *KP*-O versus *KP*-Y primary cultures. Overlapping enriched pathways between both analyses are shown in bold (*n* = 8). **b**, GSEA enrichment plot showing upregulation of UPR in *KP*-O compared with *KP*-Y identified in **a**. **c**, ISR pathway schematics. **d**, Western blot of *KP*-Y and *KP*-O in high- and low-glucose (Glc) media for the indicated time points after media renewal. **e**, Heatmap of ATF4 target gene expression in *KP*-Y and *KP*-O (Human Gene Set IGARASHI_ATF4_TARGETS_DN). **f**, Western blot of *KP*-Y and *KP*-O showing ATF4 protein levels measured after addition of 50 ng ml^−^^1^ actinomycin D (ActD) (top), 10 µM MG-132 (middle) and combination of actinomycin D and MG-132 (bottom) at indicated time points. **g**, Quantification of ATF4-positive nuclei in lung tumours from *KP*-Young (*n* = 72 tumours) and *KP*-Old (*n* = 55 tumours). Representative ATF4 IHC images are shown. Data are median. Two-sided Kolmogorov–Smirnov test. ER, endoplasmic reticulum; FC, fold change; FDR, false discovery rate; NES, normalized enrichment score. Scale bars, 20 µm. Source data

**Extended Data Fig. 4:**
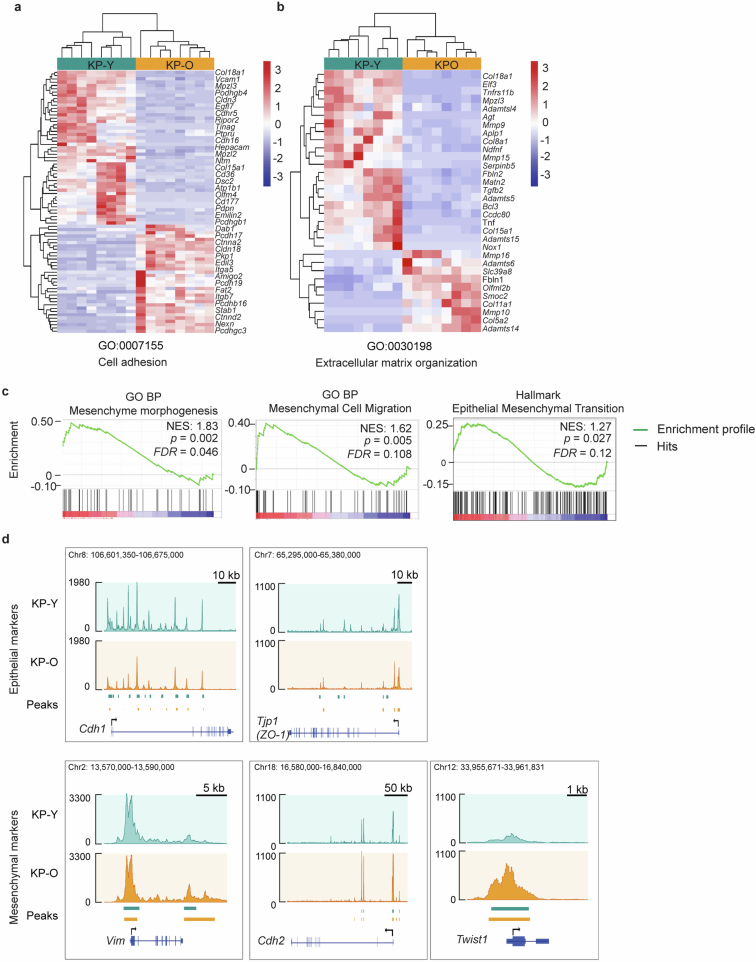
Metastasis-related pathways are enriched in *KP*-O primary cultures. **a-b**, Heatmap of GO-BP genes related to cell adhesion (every other gene shown) (**a**) and extracellular matrix reorganization (**b**) in *KP*-Y and *KP*-O (*n* = 8) primary cultures. Expression values for each gene across all samples were normalized by z-score **c**, GSEA enrichment plot for Mesenchymal morphogenesis, Mesenchymal cell migration and Epithelial mesenchymal transition in *KP*-O compared to *KP*-Y; FDR, false discovery rate; NES, normalized enrichment score. **d**, ATAC-seq genome browser view showing chromatin accessibility of indicated epithelial *Cdh1* (E-CADHERIN) and *Tjp1* (ZO-1) (top) and mesenchymal *Vimentin*, *Cdh2* (N-CADHERIN), and *Twist1* (bottom) markers in *KP*-Y and *KP*-O. Identified peaks in *KP*-Y and *KP*-O are shown below the tracks, in green and orange respectively (*n* = 6). Statistical significance was determined by permutation-based GSEA using a weighted Kolmogorov–Smirnov–like statistic with FDR correction (**c**). Statistical significance was assessed by using Diffbind with DeSeq2 (**d**).

**Extended Data Fig. 5:**
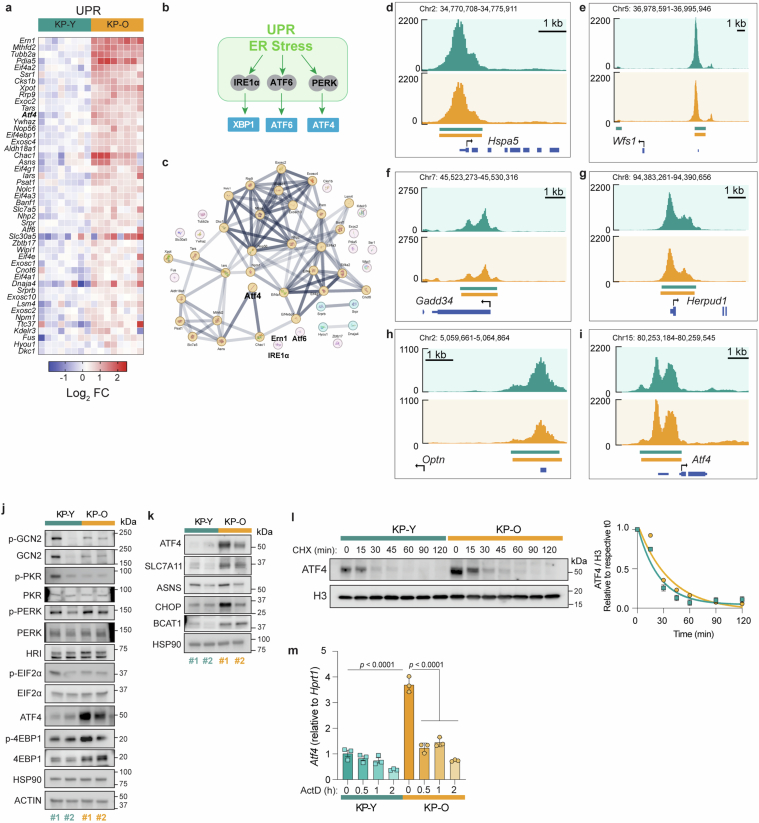
ISR effector ATF4 and targets are enriched in *KP*-O primary cultures. **a**, Heatmap of core enriched genes from unfolded protein response (UPR) pathway identified by GSEA indicating the Log2FC expression in *KP*-Y and *KP*-O primary cultures (*n* = 8). **b**, Schematic of UPR pathway. **c**, STRING protein–protein interaction network of UPR genes shown in (**a**), highlighting an ATF4-centered module (p < 1.1016). Main sensors of the UPR pathway IRE1α (*Ern1*), ATF6 and ATF4 are shown. **d-i**, ATAC-seq tracks showing the changes in chromatin accessibility of UPR chaperone *Hspa5* (**d**), UPR termination Wfs1 (**e**), ISR negative regulator Gadd34 (**f**), UPR resolution *Herpud1* and *Optn* (**g,h**), and *Atf4* (**i**). Identified peaks in *KP*-Y and *KP*-O are shown below the tracks, in green and orange respectively (*n* = 3). **j**, Western Blot analysis of *KP*-Y and *KP*-O showing ATF4 and ATF4 upstream regulators. **k**, Western blot analysis of *KP*-Y and *KP*-O showing ATF4 and ATF4 downstream regulators. **l**, Western blot analysis of *KP*-Y and *KP*-O showing ATF4 protein levels measured at the indicated times after addition of 20 µg/ml cycloheximide (CHX). Ratio of ATF4 levels to loading control H3 represented at each time point relative to their respective level at time zero in *KP*-Y and *KP*-O (*n* = 3). **m**, Relative mRNA expression of *Atf4* at the indicated times after addition of 50 ng/ml Actinomycin D (ActD) in *KP*-Y and *KP*-O (*n* = 3) primary cultures. Data are mean values ± s.e.m. Statistical significance was assessed by using Diffbind with DeSeq2 (**d-i**), Ordinary one-way ANOVA with Tukey’s multiple comparisons test (**m**).

Age-associated cellular changes are often regulated through epigenetic alterations in chromatin accessibility^17–20^. To elucidate mechanisms driving the increased metastatic propensity of lung tumours from old mice, we performed unbiased RNA sequencing (RNA-seq) and assay for transposase-accessible chromatin using sequencing (ATAC-seq) of *KP*-Y and *KP*-O cultures, identifying 2,022 differentially expressed genes (DEGs) and 27,206 differentially accessible chromatin regions in *KP*-O compared with *KP*-Y cultures. Pathway enrichment analyses (gene set enrichment analysis (GSEA), RNA-seq and Genomic Regions Enrichment of Annotations Tool (GREAT), ATAC-seq) identified key pathways associated with transcriptional and epigenetic alterations. Among the top eight enriched pathways, two dominant and overlapping themes emerged: EMT and the unfolded protein response (UPR) (Fig. 2a and Supplementary Table 1), both highly enriched in *KP*-O cultures (Fig. 2b, Extended Data Figs. 4a–d and 5a and Supplementary Table 2).

The UPR signals endoplasmic reticulum stress through three branches: IRE1α (encoded by *ERN1*)–XBP1, ATF6 and PERK–eIF2α–ATF4 (Extended Data Fig. 5b). STRING analysis of *KP*-O culture-enriched DEGs revealed a significant enrichment for an ATF4-including network (Extended Data Fig. 5c), whereas IRE1α and ATF6 lacked any functional associations, strongly suggesting a predominant UPR–PERK–ATF4 activation in *KP*-O cultures. The UPR feeds into the integrated stress response (ISR) by means of PERK-mediated phosphorylation of eIF2α, converging on induction of ATF4, the master ISR transcriptional effector^21^ (Fig. 2c).

To explore how ageing modulates these pathways, primary cultures were exposed to low-glucose conditions for 6–24 hours, a physiologically relevant nutrient stress encountered by disseminating tumour cells also known to induce endoplasmic reticulum stress. Under low-glucose conditions, *KP*-O cultures showed markedly stronger activation of the PERK branch of the UPR–ISR, with increased phosphorylation of PERK (p-PERK), eIF2α (p-eIF2α) and elevated ATF4 protein levels compared with *KP*-Y cultures (Fig. 2c,d). Notably, in *KP*-O cultures, p-eIF2α levels remained elevated following glucose deprivation, coinciding with ATF4 induction. However, ATF4 protein expression persisted even after p-eIF2α levels declined due to feedback regulation by means of GADD34 (Fig. 2c,d). Even minor environmental fluctuations, such as media replacement under standard high glucose, triggered an increase of p-eIF2α (Fig. 2d), revealing a lower threshold for ISR activation in *KP*-O cultures.

ATAC-seq revealed increased chromatin accessibility at the *Atf4* locus in *KP*-O cultures, consistent with enhanced transcriptional activity and rapid stress-induced ATF4 induction, alongside reduced accessibility at several UPR-resolving loci (*Hspa5*, *Wfs1*, *Gadd34* (*Ppp1r15a*), *Herpud1* and *Optn*)^22–26^ (Extended Data Fig. 5d–i). Reduced accessibility at these loci indicates impaired stress resolution, providing a mechanistic basis for the sustained and elevated ATF4 signalling observed in *KP*-O cultures. Accordingly, ATF4 emerged as a central age-regulated hub, with an ATF4 gene signature differentially expressed in *KP*-O compared with *KP*-Y cultures (Fig. 2e and Supplementary Table 1). Together, these findings suggest that ageing sensitizes tumour cells to endoplasmic reticulum stress by amplifying PERK–ATF4-mediated signalling.

We noted that 48 hours after seeding in normal culture conditions, despite similar phosphorylation levels of the main upstream ISR kinases (GCN2, PKR, PERK, HRI) and ISR core protein eIF2a^27^ (Fig. 2c and Extended Data Fig. 5j), ATF4 protein and downstream targets, including p-4EBP1, ASNS, SLC7A11, CHOP and BCAT1, remained markedly higher in *KP*-O cultures (Extended Data Fig. 5j,k). ATF4 protein stability was comparable between *KP-*O and *KP*-Y cultures (Extended Data Fig. 5l). Whereas *Atf4* mRNA decayed more rapidly in *KP*-O cultures on transcriptional inhibition (Extended Data Fig. 5m), ATF4 protein was rapidly depleted following transcriptional inhibition in both *KP*-Y and *KP*-O (Fig. 2f). By contrast, proteasomal inhibition led to greater ATF4 accumulation in *KP*-O cultures (Fig. 2f), consistent with increased transcription and/or translation, but failed to restore ATF4 protein levels when transcription was blocked. These data indicate that the elevated ATF4 levels in *KP*-O cultures primarily reflects increased transcription rather than alterations in mRNA stability or protein turnover. Collectively, these findings indicate that increased transcription through permissive chromatin states and amplified ISR signalling act together to drive the sustained and elevated ATF4 levels observed in *KP*-O cultures.

## ATF4 drives ageing-induced metastasis

**Fig. 3:**
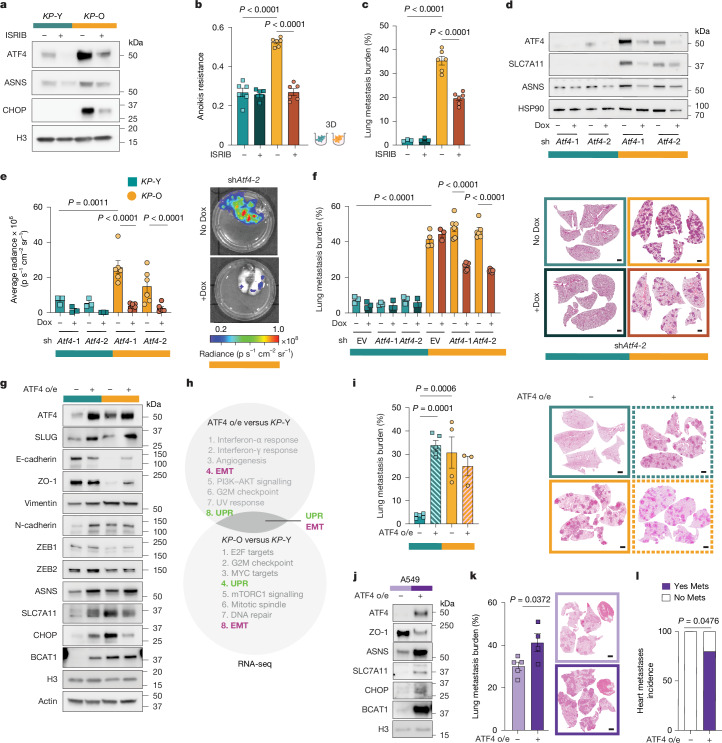
ATF4-induced plasticity is necessary and sufficient for driving metastasis. **a**, Western blot of *KP*-Y and *KP*-O primary cultures treated with 2 μM ISRIB twice daily for 72 h. **b**, Anoikis resistance of *KP*-Y and *KP*-O treated with 2 µM ISRIB (*n* = 6). **c**, Lung metastatic burden 7 days after intravenous injections of *KP*-Y (*n* = 3 per group) and *KP*-O (*n* = 6 per group). **d**, Western blot analysis of ATF4 and ATF4 targets in *KP*-Y and *KP*-O expressing a doxycycline (Dox)-inducible sh*Atf4*. **e**,**f**, Lung metastatic burden 12 days after intravenous injections of *KP*-Y (*n* = 3) and *KP*-O (*n* = 6) expressing Dox-inducible sh*Atf4* measured by means of bioluminescence (**e**), or H&E quantification (*KP*-Y with or without Dox (*n* = 3), *KP*-O EV (*n* = *4*), *KP*-O EV + Dox (*n* = 3), *KP*-O with or without Dox (*n* = 6)) (**f**). **g**, Western blot analysis of ATF4, ATF4 targets and EMT markers in *KP*-Y and *KP*-O with or without ATF4 overexpression (o/e). **h**, Top GSEA enriched pathways in *KP*-O versus *KP*-Y and *KP*-Y ATF4 o/e versus *KP*-Y. In bold, overlapping enriched pathways. **i**, Lung metastatic burden and representative H&E-stained lung sections following intravenous injections of *KP*-Y and *KP*-O expressing control or ATF4 o/e (*n* = 5, 5, 4, 3). **j**, Western blot analysis of ATF4, ATF4 targets and EMT marker in A549 control and ATF4 o/e cells. **k**, Lung metastatic burden and representative H&E-stained lung sections following intravenous injections of A549 expressing either control or o/e ATF4 (*n* = 5, 4). **l**, Heart metastasis incidence in mice injected with A549 control or ATF4 o/e cells (*n* = 5). Data are mean values ± s.e.m. Ordinary one-way ANOVA with Tukey’s multiple comparisons test (**b**,**c**,**e**,**f**,**i**), two-sided unpaired *t*-test (**k**) and two-sided Fisher’s exact test (**l**). Scale bars, 1 mm (**f**,**i**,**k**). Source data

**Extended Data Fig. 6:**
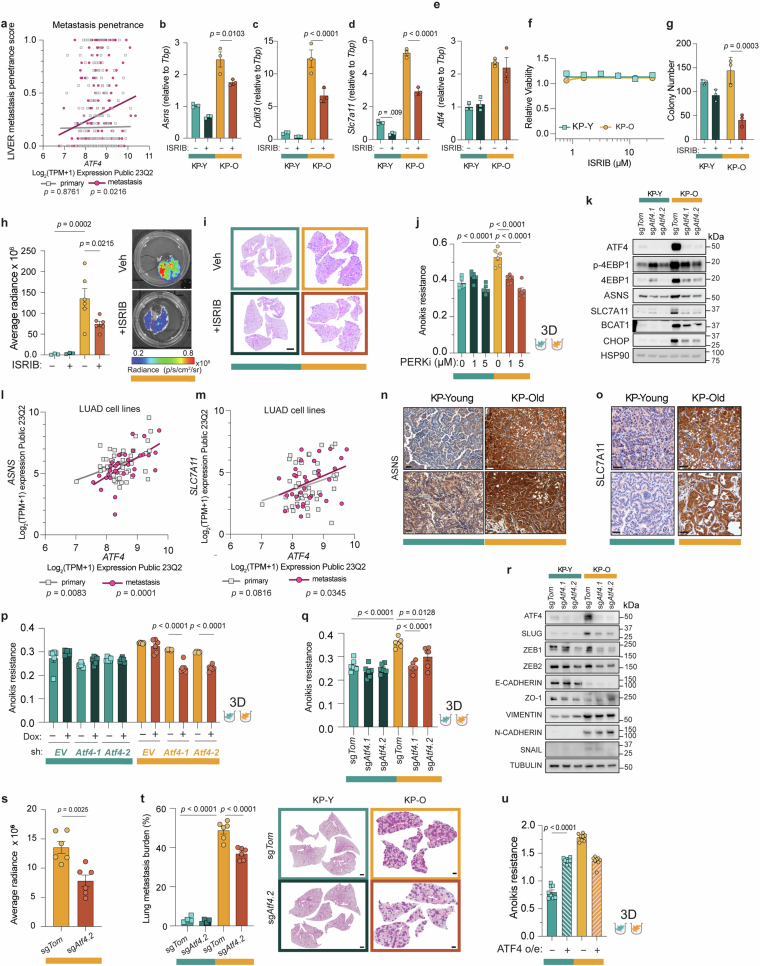
EMT, anoikis and metastasis are dependent on ATF4 in *KP*-O primary cultures. **a**, Correlation between *ATF4* expression and Liver metastasis penetrance in human cell lines from DepMap (*n* = 462). **b-e**, Relative mRNA expression of *Atf4* and its targets *Asns, Ddit3, Slc7a11* following twice daily treatment with 2 µM ISRIB for 72 h in *KP*-Y and *KP*-O primary cultures (*n* = 3). **f**, *KP*-Y and *KP*-O were treated twice daily with 2 µM ISRIB for 72 h and relative viability assessed after another 72 h. **g**, Colony number of *KP*-Y (*n* = 3) and *KP*-O (*n* = 3) grown with 2 µM ISRIB for 12 days (*n* = 3). **h-i**, Lung metastasis burden after intravenous injections of *KP*-Y (*n* = 3) and *KP*-O (*n* = 6) in mice and treated with either vehicle or 10 mg/kg ISRIB i.p twice daily, measured via bioluminescence (and representative ex vivo bioluminescence images of lungs, **h**). Representative H&E-stained lung sections (scale bars, 2 mm, **i**). H&E-quantification shown in Fig. 3c. **j**, Anoikis resistance of *KP*-Y and *KP*-O (*n* = 6) treated with 1 µM or 5 µM PERK inhibitor (PERKi). **k**, Western blot of ATF4 and ATF4 targets in sg*Tom* and *Atf4*-KO *KP*-Y and *KP*-O. **l-m**, Correlation between *ATF4* expression and ATF4 target gene *ASNS* (**l**) and *SLC7A11* (**m**) in human lung adenocarcinoma (LUAD) cell lines from Dependency Map (DepMap) portal (*n* = 77). **n-o**, IHC staining of ASNS (**n**) and SLC7A11 (**o**) in lung tumors from *KP*-Young and *KP*-Old mice 21 weeks after tumor initiation with Lenti-Cre (Scale bars, 50 µm). **p-q**, Anoikis resistance of *KP*-Y and *KP*-O (*n* = 6) expressing either Doxycycline (dox) inducible mirE_sh*Atf4* (**p**) or either sg*Tom* or sg*Atf4* (**q**). **r**, Western blot analysis of ATF4 and EMT markers in *KP*-Y and *KP*-O expressing either control sg*Tom* or sg*Atf4*. **s-t**, Lung metastasis burden 14 days after intravenous injection of *KP*-Y and *KP*-O expressing either sg*Tom* (*KP*-Y sg*Tom n* = 4; *KP*-O sg*Tom n* = 4) or sg*Atf4* (*KP*-Y sg*Atf4 n* = 4; *KP*-O sg*Atf4 n* = 6). Scale bars, 1 mm. **u**, Anoikis resistance of *KP*-Y and *KP*-O expressing either control or ATF4 o/e (*n* = 8). Data are mean values ± s.e.m. Simple linear regression (**a,l,m**), Ordinary one-way ANOVA with Sidak’s multiple comparisons test (**b-e**), Ordinary one-way ANOVA with Tukey’s multiple comparisons test (**g,h, j,p,q,t,u**) and two-sided unpaired t-test (**s**). Source data

ATF4 has been implicated in anoikis resistance and metastasis^28,29^ and its expression strongly correlates with liver metastasis penetrance of human cancer cell lines (Extended Data Fig. 6a). Consistently, the percentage of ATF4-positive nuclei was markedly higher in tumours from *KP*-Old mice compared with *KP*-Young (Fig. 2g). To determine whether ATF4 has a causal role in ageing-induced metastasis, we first pharmacologically inhibited ATF4 translation using inhibitor of integrated stress response (ISRIB)^30^, an ISR inhibitor that reduced ATF4 protein levels and its downstream target genes in *KP*-O cultures (Fig. 3a and Extended Data Fig. 6b–e). Chronic ISRIB treatment, without affecting adherent cells viability, decreased anoikis resistance, colony-forming ability and in vivo metastatic propensity of *KP*-O cultures (Fig. 3b,c and Extended Data Fig. 6f–i). Similarly, PERK inhibition decreased anoikis resistance (Extended Data Fig. 6j), supporting a role for the PERK–ATF4 axis in this ageing-induced phenotype. We next targeted ATF4 genetically by CRISPR–Cas9-mediated deletion (single-guide RNAs targeting *Atf4*) (Extended Data Fig. 6k) and doxycycline-inducible mirE-based short-hairpin RNA (shRNA)-mediated depletion of ATF4 (Fig. 3d). Protein levels of ATF4 and downstream targets, including ASNS, SLC7A11 and 4EBP1, were lowered on both approaches (Fig. 3d and Extended Data Fig. 6k) in both *KP*-Y and *KP*-O cultures. Of note, ASNS and SLC7A11 expression, but not 4EBP1 expression, strongly correlated with ATF4 expression in human lung adenocarcinoma (LUAD) cell lines, suggesting that the first two could be involved in ATF4-driven metastasis (Extended Data Fig. 6l,m). Supporting this, tumours from *KP*-Old mice stained strongly positive for both ASNS and SLC7A11 (Extended Data Fig. 6n,o). Furthermore, knockout or knockdown of *Atf4* reduced anoikis resistance and fitness in *KP*-O but not *KP*-Y cultures (Extended Data Fig. 6p,q) and significantly downregulated EMT markers (Extended Data Fig. 6r). To confirm whether the increased metastatic capacity of tumour cells from *KP*-O mice was ATF4-dependent in vivo, *KP*-O and *KP*-Y cultures with ATF4 depletion or deletion were transplanted by tail-vein injections. Loss or knockdown of *Atf4* in *KP*-O cultures significantly reduced lung metastasis burden compared with non-targeted *KP*-O controls (Fig. 3e,f and Extended Data Fig. 6s,t). By contrast, mice injected with *KP*-Y cultures showed very little lung metastasis burden compared with *KP*-O injected mice, regardless of ATF4 targeting (Fig. 3e,f and Extended Data Fig. 6s,t). Overall, these results indicate that ATF4 regulates ageing-induced metastatic potential in primary *KP*-O cultures.

To assess whether ATF4 is sufficient to drive metastasis, we overexpressed ATF4 in *KP*-Y and *KP*-O cultures (Fig. 3g). ATF4 overexpression led to similarly modest increase in ATF4 levels in both *KP*-Y and *KP*-O, along with upregulation of ATF4 target genes encoding ASNS, SLC7A11, CHOP and BCAT1. Notably, EMT marker proteins were also induced on ATF4 overexpression in *KP*-Y cultures (Fig. 3g). ATF4 overexpression in *KP*-Y cultures led to the differential expression of 1,661 genes, with an overlap of 592 DEGs between ATF4-overexpressing *KP*-Y and *KP*-O versus *KP*-Y transcriptomes. GSEA identified enrichment for UPR and EMT pathways in the ATF4 overexpression (Fig. 3h and Supplementary Table 1) similar to the enrichment observed in *KP*-O, reinforcing the role of ATF4 in ageing-induced metastasis. Functionally, ATF4 overexpression in *KP*-Y increased both anoikis resistance and metastasis to similar levels as in *KP*-O (Fig. 3i and Extended Data Fig. 6u). ATF4 overexpression in human A549 lung adenocarcinoma cells was sufficient to markedly increase lung metastatic burden and enable colonization of extrapulmonary sites such as the heart (Fig. 3j–l). Thus, ATF4 is both necessary and sufficient to confer the metastatic state induced by ageing.

## ATF4-driven metabolic rewiring in ageing

**Fig. 4:**
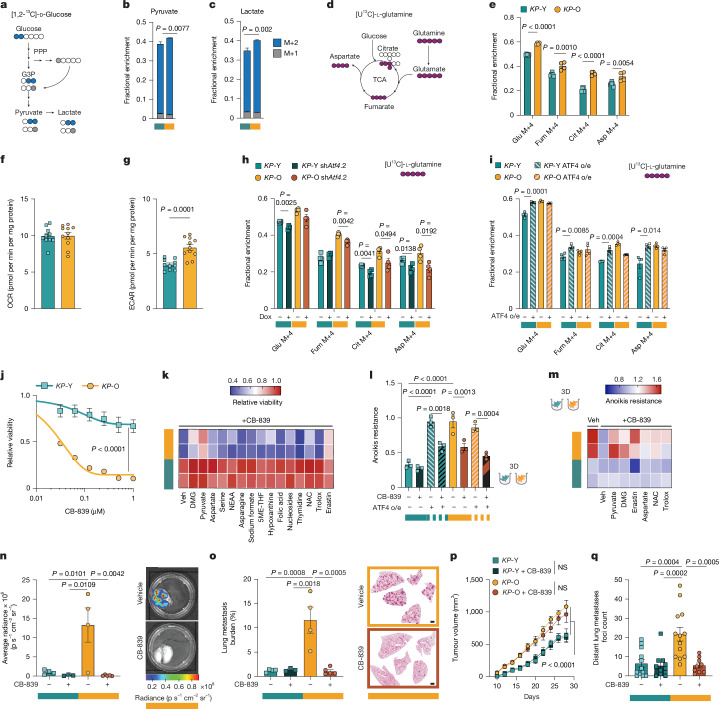
Targeting ATF4-induced metabolic plasticity blocks metastasis. **a**, Schematic of [1,2-^13^C]-d-glucose tracing in glycolysis (blue circles) or the pentose phosphate pathway (PPP) (grey circles). **b**,**c**, Fractions of labelled pyruvate (**b**) and lactate (**c**) from [1,2-^13^C]-d-glucose in *KP*-Y and *KP*-O (*n* = 6). **d**, Schematic of [U^13^C]-l-glutamine tracing (pink circles). **e**, Mass isotopomer analysis of glutamate (Glu), fumarate (Fum), citrate (Cit) and aspartate (Asp) from [U^13^C]-l-glutamine tracing in *KP*-Y (*n* = 6) and *KP*-O (*n* = 4). **f**,**g**, Oxygen consumption rate (OCR) (**f**) and extracellular acidification rate (ECAR) (**g**) in *KP*-Y (*n* = 10) and *KP*-O (*n* = 11). **h**,**i**, Mass isotopomer analysis of indicated TCA intermediates from [U^13^C]-l-glutamine tracing in *KP*-Y, *KP*-O, *KP*-Y sh*Atf4*.2,* KP*-O sh*Atf4*.2 (**h**) and in *KP*-Y; *KP*-Y ATF4 o/e; *KP*-O and *KP*-O ATF4 o/e (**i**) (*n* = 4 per condition). **j**, Relative viability of *KP*-Y and *KP*-O treated with CB-839 (*n* = 3) **k**, Heatmap of relative viability for *KP*-Y and *KP*-O treated with 0.1 μM CB-839 and the indicated compounds. All data points are relative to vehicle (Veh)-treated controls (*n* = 3). **l**, Anoikis resistance of *KP*-Y; *KP*-Y ATF4 o/e; *KP*-O and *KP*-O ATF4 o/e treated with 0.1 μM CB-839 for 48 h (*n* = 3). **m**, Heatmap of anoikis resistance of *KP*-Y and *KP*-O treated with 0.1 μM CB-839 and the indicated compounds. All data points are relative to vehicle-treated controls (*n* = 3). **n**,**o**, Lung metastasis burden measured by means of bioluminescence (**n**) or H&E quantification (**o**) (with representative images) in mice after intravenous injections of *KP*-Y and *KP*-O and treated with CB-839 or vehicle (*n* = 4, 3, 4, 5). **p**,**q**, Longitudinal tumour growth of subcutaneous tumours (*KP*-Y *n* = 24, *KP*-Y + CB-839 *n* = 22, *KP*-O *n* = 26 and *KP*-O + CB-839 *n* = 18 tumours) (**p**) and H&E quantification of lung metastases foci (*n* = 12, 11, 13 and 9 mice, respectively) (**q**). Mice were administered CB-839 once the tumours reached 100 mm^3^ in size. Data are mean ± s.e.m. Two-sided multiple unpaired *t*-tests (**b**,**c**,**e**–**i**,**q**), two-way ANOVA (**j**,**p**), one-way ANOVA with Tukey’s multiple comparisons test (**l**,**n**,**o**). 5ME-THF, 5-methyltetrahydrofolate; NAC, *N*-acetyl-l-cysteine; NEAA, non-essential amino acids; NS, not significant. Scale bars, 1 mm. Source data

**Extended Data Fig. 7:**
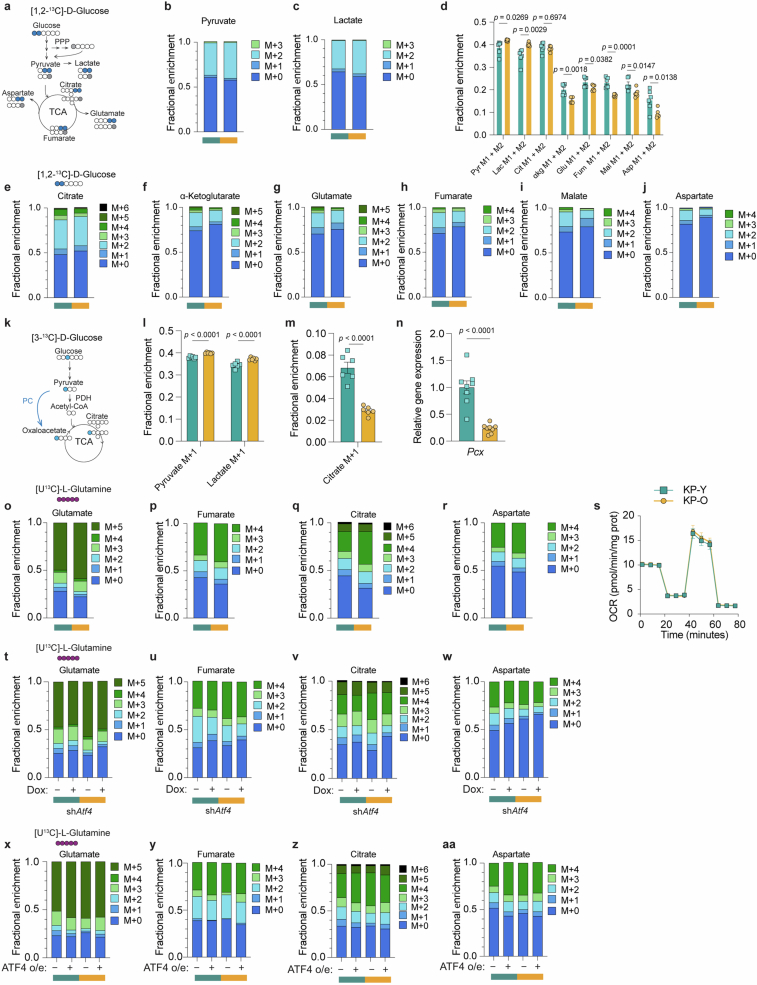
Glucose and glutamine tracing reveals metabolic differences in *KP*-Y and *KP*-O primary cultures. **a**, Schematic depicting the fate of 13 C atoms (blue circles) from [1,2-^13^C]-D-Glucose tracing. **b-c**, Fractions of pyruvate (**b**) and lactate (**c**) derived from [1,2-^13^C]-D-Glucose in *KP*-Y and *KP*-O primary cultures (*n* = 6). **d**, Mass isotopomer analysis (m1 + m2) of pyruvate, lactate and TCA cycle intermediates derived from [1,2-^13^C]-D-Glucose stable isotope tracing from *KP*-Y and *KP*-O (*n* = 6). **e-j**, Fractions of citrate (**e**), α-Ketoglutarate (**f**), glutamate (**g**), fumarate (**h**), malate (**i**) and aspartate (**j**) isotopomers derived from [1,2-^13^C]-D-Glucose in *KP*-Y and *KP*-O (*n* = 6). **k**, Schematic showing the metabolism of isotope-labelled [3-^13^C]-D-Glucose via pyruvate carboxylase (PC) or pyruvate dehydrogenase (PDH) pathways. Blue circles represent ^13^C atoms derived from [3-^13^C]-D-Glucose. (**l-m**), Mass isotopomer analysis of pyruvate and lactate (**l**), and citrate (**m**) from 8 h [3-13 C]-D-Glucose tracing in *KP*-Y and *KP*-O (*n* = 6). **n**, *Pcx* gene expression levels (*n* = 8). **o-r**, Fractions of glutamate (**o**), fumarate (**p**), citrate (**q**) and aspartate (**r**) isotopomers derived from [U^13^C]-L-Glutamine in *KP*-Y (*n* = 6) and *KP*-O primary cultures (*n* = 4). **s**, Oxygen consumption rate (OCR) measurements using the Seahorse Cell Mito Stress Test in *KP*-Y and *KP*-O primary cultures (*n* = 10 and 11). **t-aa**, Fractions of glutamate (**t**), fumarate (**u**), citrate (**v**) and aspartate (**w**) isotopomers from [U^13^C]-L-Glutamine in *KP*-Y, *KP*-Y sh*Atf4* and *KP*-O,* KP*-O shAtf4 primary cultures (*n* = 4) (**t-w**) and in control or ATF4 overexpression (o/e) in *KP*-Y and *KP*-O (**x-aa**) (*n* = 4). Data are mean values ± s.e.m. Two-sided unpaired t-test (**d, l-n**).

To investigate whether ATF4, a master regulator of metabolic stress response and cellular metabolism^21^, alters metabolism in *KP*-O cultures, we performed stable isotope tracing with [1,2-^13^C]-d-glucose. *KP*-O cultures showed increased glycolytic flux, evidenced by higher labelling of pyruvate and lactate produced through glycolysis but not the pentose phosphate pathway, which supports metastatic cancer cells by maintaining redox homeostasis^31,32^ (Fig. 4a–c and Extended Data Fig. 7a–c). By contrast, glucose-derived anaplerotic input into the tricarboxylic acid (TCA) cycle was reduced in *KP*-O cultures (Extended Data Fig. 7a,d–j). Glucose anaplerosis enters the TCA cycle through the pyruvate dehydrogenase pathway, supporting energy production or the pyruvate carboxylase pathway, which replenishes biosynthetic intermediates^33^. Tracing with [3-^13^C]-glucose showed significantly reduced pyruvate carboxylase-mediated carbon incorporation in *KP*-O cultures (Extended Data Fig. 7k–n), suggesting a metabolic shift away from glucose anaplerosis. We therefore proposed that *KP*-O cells might show metabolic plasticity by relying more heavily on alternative metabolic pathways to maintain TCA cycle intermediates. Supporting this, stable isotope tracing with [U^13^C]-l-glutamine revealed increased glutamine-derived anaplerosis in *KP*-O compared with *KP*-Y cultures (Fig. 4d,e and Extended Data Fig. 7o–r). This metabolic shift was further supported by the marked increase in extracellular acidification rates in *KP*-O versus *KP*-Y, without any change in oxygen consumption ratess, in line with increased aerobic glycolysis and glutamine anaplerosis (Fig. 4f,g and Extended Data Fig. 7s). Glutamine contribution to TCA intermediates was markedly reduced in *Atf4-*deficient *KP*-O cultures (Fig. 4h and Extended Data Fig. 7t–w) and significantly increased following ATF4 overexpression in *KP*-Y cultures (Fig. 4i and Extended Data Fig. 7x-aa), indicating that elevated glutaminolysis is an ATF4-dependent feature in *KP*-O cultures. Altogether, these results show that ATF4 rewires metabolism of *KP*-O cultures to favour metastasis.

## Targeting metabolic plasticity

**Extended Data Fig. 8:**
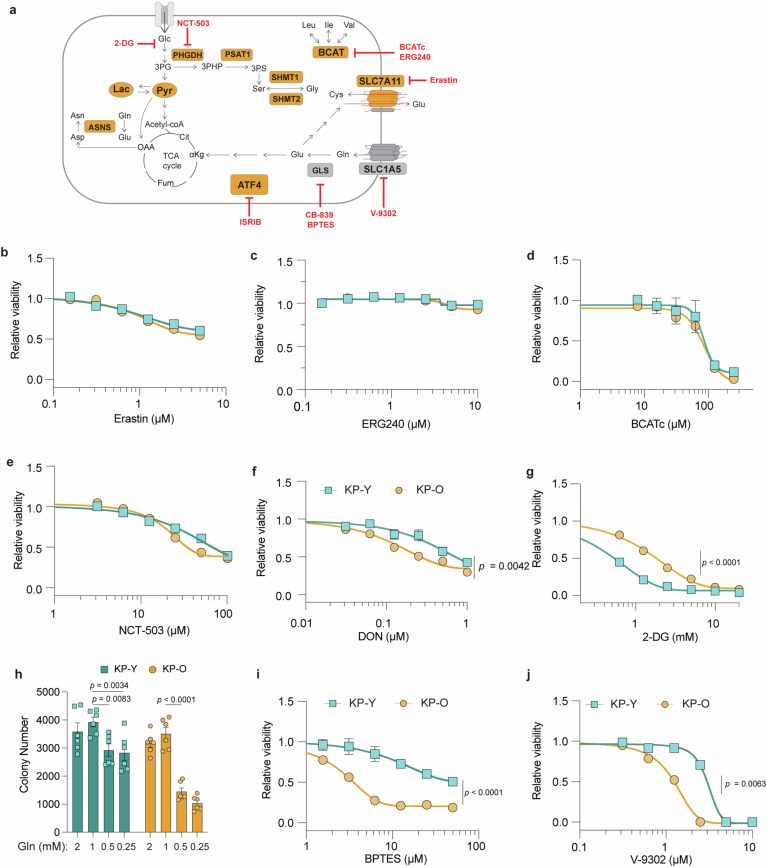
ATF4-directed drug screening identifies sensitivity to glutaminase inhibition in *KP*-O primary cultures. **a**, Schematics showing the mode of action of the inhibitors used in *KP*-Y and *KP*-O primary cultures. The enzymes and metabolites depicted in orange are ATF4 targets increased in *KP*-O. **b-g**, Relative viability assessed by CellTiter-Glo luminescence of *KP*-Y and *KP*-O after 72 h treatment with increasing doses of Erastin (**b**), ERG240 (**c**), BCATc (**d**), NCT-503 (**e**) DON (**f**), or 2-DG (**g**). All values were normalized to their respective vehicle-treated control (*n* = 3). **h**, Colony number of *KP*-Y and *KP*-O grown in RPMI media with indicated L-Glutamine (Gln) concentrations for 6 days (*n* = 6). **i-j**, Relative viability assessed by CellTiter-Glo luminescence of *KP*-Y and *KP*-O after 72 h treatment with increasing doses of BPTES (**i**) or V-9302 (**j**). All values were normalized to their respective vehicle-treated control (*n* = 3). Data are mean values ± s.e.m. Two-way ANOVA (**b-g,i,j**), one-way ANOVA with Tukey’s multiple comparisons test (**h**).

We next evaluated whether ATF4-driven metabolic plasticity in *KP*-O cultures could be therapeutically exploited (Extended Data Fig. 8a). Initial drug screening revealed no differential sensitivity to inhibitors of SLC7A11, BCAT or PHGDH (Extended Data Fig. 8b–e). However, *KP*-O cultures were more sensitive to glutamine depletion and to the glutamine analogue DON, but not to the glycolysis inhibitor 2-deoxy-glucose (2-DG) (Extended Data Fig. 8f–h). We therefore tested GLSi that block the rate-limiting conversion of glutamine into glutamate, known as glutaminolysis (Extended Data Fig. 8a). *KP*-O cultures showed a marked sensitivity to two independent GLSi, CB-839 (telaglenastat) and BPTES (Fig. 4j and Extended Data Fig. 8i), consistent with previous links between high SLC7A11 levels and GLS inhibition sensitivity^34,35^. In addition, *KP*-O cultures were more sensitive than *KP*-Y cultures to V-9302, an antagonist of the main glutamine transporter ASCT2 (Extended Data Fig. 8j).

**Extended Data Fig. 9:**
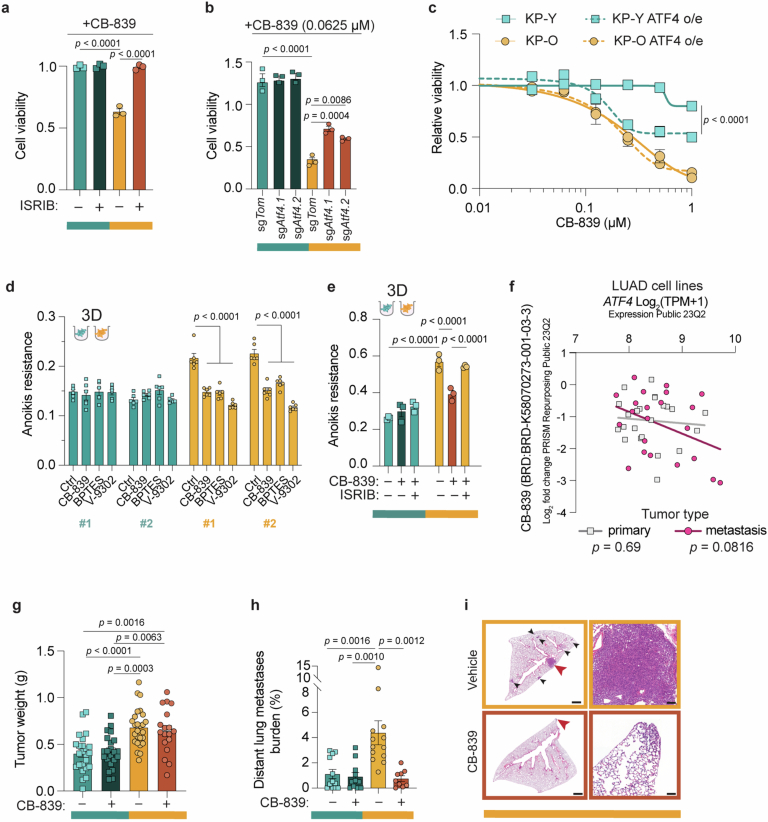
*KP*-O sensitivity to glutamine targeting is dependent on ATF4. **a**, Cell viability measured by CellTiter-Glo of *KP*-Y and *KP*-O primary cultures treated for 72 h with 1 µM ISRIB followed by 0.1 µM CB-839 treatment for another 72 h. All values were normalized to their respective vehicle-treated control (*n* = 3). **b**, Cell viability measured by CellTiter-Glo of *KP*-Y and *KP*-O (sg*Tom* or sg*Atf4)* treated with 0.0625 µM CB-839 for 72 h. All values were normalized to their respective vehicle-treated control (*n* = 3). **c**, Relative viability assessed by CellTiter-Glo of *KP-*Y and *KP*-O expressing either control or ATF4 o/e following 0.1 µM CB-839 treatment for 72 h. All values were normalized to their respective vehicle-treated control (*n* = 3). **d**, Anoikis resistance of *KP*-Y and *KP*-O treated with either vehicle control, 0.1 µM CB-839, 5 µM BPTES or 2 µM V9302 (*n* = 6). **e**, Anoikis resistance of *KP*-Y and *KP*-O treated twice a day for 72 h with 2 μM ISRIB and then seeded in ULA plates with either vehicle, 0.1 µM CB-839, 2 µM ISRIB or a combination of both (*n* = 3). **f**, Correlation analysis between *ATF4* expression and CB-839 sensitivity in human lung adenocarcinoma (LUAD) cell lines (*n* = 45) from Dependency Map (DepMap) portal. Primary tumor cell lines are depicted with grey squares, metastatic cell lines with pink circles. **g-i**, *KP*-Y and *KP*-O subcutaneously injected in mice. Mice were administered 200 mg/kg CB-839 p.o twice/day every other day for the duration of the experiment once the tumors reached 100 mm^3^ in size. Complementary results presented in Fig. 4p and Fig. 4q. **g**, Endpoint tumor weight after subcutaneous injections of *KP*-Y and *KP*-O. **h**, Lung metastasis burden. **i**, Left, H&E- stained lung sections showing metastatic foci in the lungs (black and red arrows). Right, close-up of the area identified by the red arrow in the left panel (Scale bars, 1 mm and 100 µm). Data are mean values ± s.e.m. Ordinary one-way ANOVA with Tukey’s multiple comparisons test **(a,b,d,e,g,h**), two-way ANOVA (**c**) and simple linear regression (**f**). Source data

To pinpoint the source of sensitivity to GLSi in *KP*-O cultures, we pretreated *KP*-O and *KP*-Y cultures with various metabolites, amino acids, antioxidants and small molecules. We found that both dimethyl-2-oxoglutarate (DMG), a cell-permeable α-ketoglutarate (α-KG) analogue and pyruvate, a glucose-derived TCA cycle carbon source, were able to rescue CB-839 sensitivity in *KP*-O cultures (Fig. 4k). CB-839 sensitivity was rescued by none of the other tested metabolites or inhibitors except the cysteine–glutamate antiporter system inhibitor erastin, strongly indicating *KP*-O cultures are highly sensitive to reduced glutamate levels (Fig. 4k). We next tested whether ATF4 mediates *KP*-O sensitivity to GLSi. Indeed, *KP*-O cultures pretreated with low doses of ISRIB, or with genetically deleted ATF4, lost their CB-839 sensitivity (Extended Data Fig. 9a,b). Accordingly, ATF4 overexpression rendered previously resistant *KP*-Y cultures sensitive to CB-839 (Extended Data Fig. 9c). In 3D cultures, *KP*-O spheroids lost their anoikis resistance on treatment with GLSi or the glutamine transporter inhibitor V-9302 (Fig. 4l,m and Extended Data Fig. 9d). Notably, spheroid sensitivity to CB-839 was ATF4-dependent, as short-term ISRIB treatment, insufficient on its own to alter anoikis resistance, rescued survival (Extended Data Fig. 9e). Furthermore, ATF4 overexpression conferred anoikis resistance to *KP*-Y spheroids, which was reversed by newly acquired CB-839 sensitivity (Fig. 4l).

Furthermore, CB-839-induced loss of anoikis resistance in *KP*-O was rescued by DMG, pyruvate and erastin but not by other interventions (Fig. 4m). Consistent with these findings, ATF4 expression correlated with CB-839 sensitivity in metastatic, but not primary, human LUAD cell lines (Extended Data Fig. 9f).

To test the in vivo relevance of our findings, *KP*-O and *KP*-Y cultures were intravenously or subcutaneously transplanted into mice and randomized to CB-839 or vehicle treatment. Following intravenous injections, *KP*-O cultures formed numerous lung metastases in vehicle-treated animals but were nearly undetectable in CB-839 treated animals (Fig. 4n,o). By contrast, *KP*-Y injected animals developed little to no metastasis regardless of treatment (Fig. 4n,o). After subcutaneous transplantation, CB-839 treatment did not affect tumour growth rates of *KP*-O and *KP*-Y (Fig. 4p). Although *KP*-O tumours were overall significantly larger than *KP*-Y tumours, final tumour weights were unchanged by CB-839 treatment within each group (Extended Data Fig. 9g). CB-839 treatment almost completely abrogated distant metastasis from *KP*-O tumours, as assessed by distant metastasis foci counts and lung metastasis burden, without affecting the growth or final size of *KP*-O tumours (Fig. 4p,q and Extended Data Fig. 9g–i). By contrast, *KP*-Y tumours and metastasis were unaffected by GLSi (Fig. 4p,q and Extended Data Fig. 9g–i). Overall, our study indicates that ageing-induced and ATF4-mediated metabolic rewiring constitutes a druggable vulnerability in the metastatic setting that could be harnessed as a potential adjuvant therapy target for older patients with NSCLC.

## Ageing alters progression of human NSCLC

**Fig. 5:**
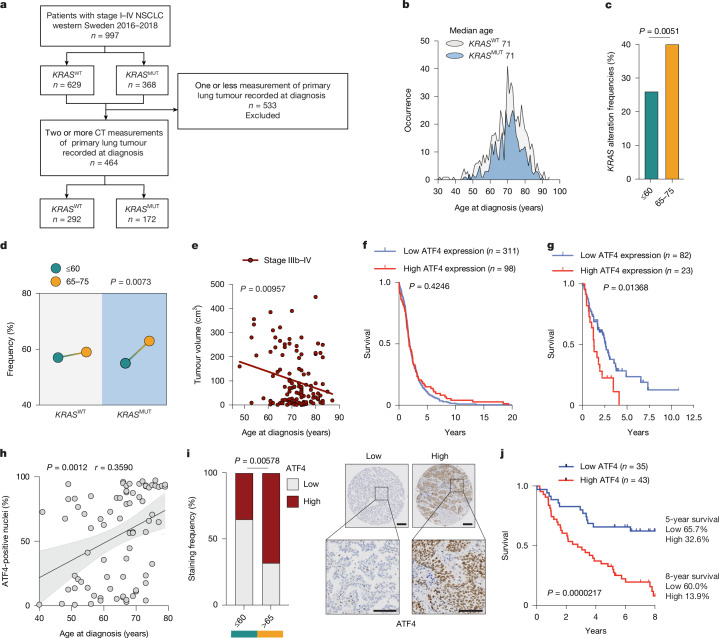
Human NSCLC recapitulates ageing phenotypes with increased metastatic risk and ATF4-linked poor outcome despite smaller primary tumours. **a**, Flow chart of patient selection for the study. **b**, Distribution of age at diagnosis for patients harbouring a *KRAS*^WT^ (light grey) or *KRAS*^MUT^ (blue) alteration. **c**, Incidence of KRAS mutations frequencies in young (60 years or younger, *n* = 124) and older (65–75 years, *n* = 476) patients at diagnosis in the western Sweden cohort. **d**, Proportion of young (60 years or younger, green circles) or older (65–75 years, orange circles) patients from the western Sweden cohort diagnosed with stage IIIb–IV (*n* = 359) NSCLC, harbouring an alteration in KRAS (*KRAS*^MUT^) or not (*KRAS*^WT^). **e**, Correlation analysis showing negative relation between tumour size and age at diagnosis in patients with stage IIIb–IV *KRAS*^MUT^ (*n* = 117) from the western Sweden cohort. **f**,**g**, Overall survival of patients with stage I–II (*n* = 409) (**f**) and patients with stage III–IV (*n* = 105) (**g**) LUAD from human protein atlas (TCGA dataset) stratified by high versus low ATF4 expression level. **h**, Correlation between percentage of ATF4-positive nuclei and age at diagnosis in patients with *KRAS*^MUT^ LUAD from the Swedish NSCLC-TMA cohort (*n* = 78), with shaded areas indicating 95% confidence intervals. **i**, ATF4 staining intensity (with representative images) in *KRAS*^MUT^ LUAD tumours from young (60 years or younger, *n* = 26) and older patients (65 years or above, *n* = 47) from the Swedish NSCLC-TMA cohort. **j**, Survival of patients with *KRAS*^MUT^ LUAD from the Swedish NSCLC-TMA cohort stratified by ATF4 staining low (*n* = 35) or high (*n* = 43). Two-sided Fisher’s exact test (**c**), two-sided Chi-square test (**d**,**i**), Simple linear regression (**e**), log-rank test (**f**,**g**,**j**) and Pearson correlation (**h**). CT, computed tomography. Scale bars, 200 μm (**i**, top) and 100 μm (**i**, bottom).

**Extended Data Fig. 10:**
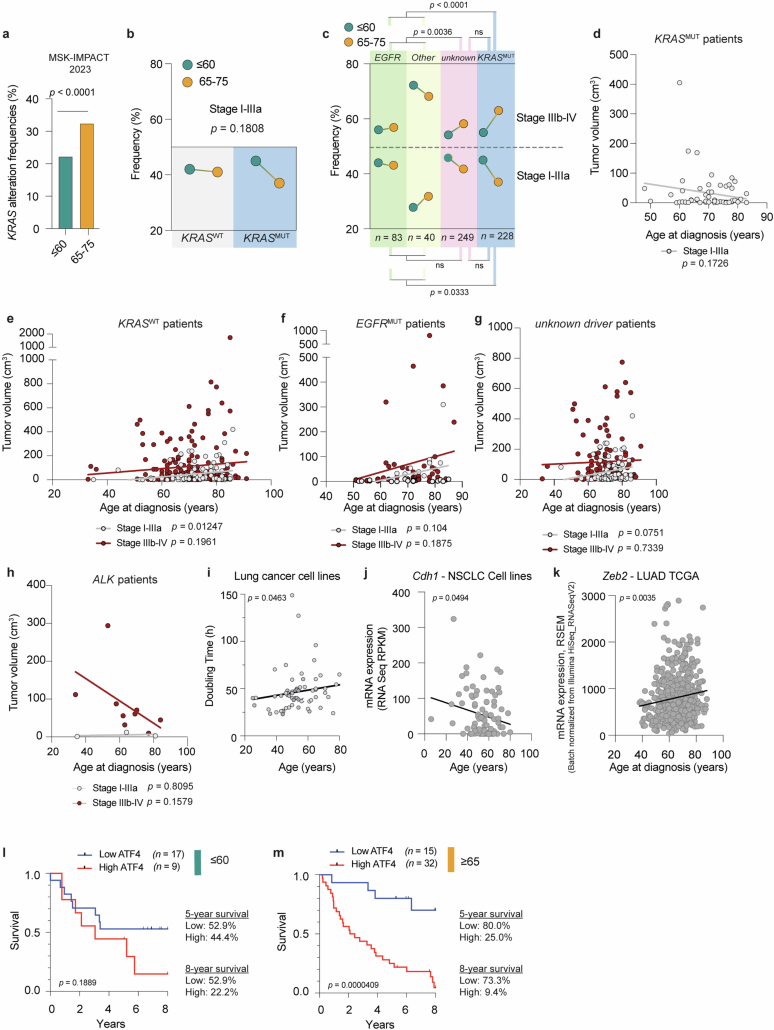
Ageing alters tumor volume and metastasis in human NSCLC. **a**, *KRAS* mutations frequencies in young (≤ 60 years) and old (65–75 years) patients at diagnosis in the MSK-IMPACT dataset (≤ 60 *n* = 145; 65–75 *n* = 290) (Lengel et al.^36^) **b**, Frequency of young (≤ 60 years, green circles) or old (65–75 years, gold circles) patients from the western Sweden cohort diagnosed with Stage I-IIIa, harboring an alteration in *KRAS* (*KRAS*^MUT^, *n* = 87) or not (*KRAS*^WT^, *n* = 154). **c**, Frequency of young (≤ 60 years, green circles) or old (65–75 years, gold circles) patients from the western Sweden cohort diagnosed with Stage I-IIIa, harboring an alteration in *EGFR* (*n* = 83), *KRAS*^MUT^ (*n* = 228), all other known drivers (*BRAF, ALK, ROS1, RET*; *n* = 40) or unknown driver mutation (*n* = 249). **d-h**, Correlation analyses between tumor size and age at diagnosis from the western Sweden cohort in *KRAS*^MUT^ patients Stage I-IIIa (*n* = 55) (**d**), *KRAS*^WT^ patients (Stage I-IIIa, *n* = 104; Stage IIIb-IV *n* = 188) (**e**), *EGFR*^MUT^ patients (Stage I-IIIa *n* = 23; Stage IIIb-IV, *n* = 45) (**f**), patients with unknown driver mutations (Stage I-IIIa *n* = 3; Stage IIIb-IV, *n* = 9) (**g**), patients with *ALK* alterations (Stage I-IIIa, *n* = 75; Stage IIIb-IV *n* = 119) (**h**). **i**, Positive correlation between doubling time (hours) of lung cancer cell lines and age (years) from EXPASY dataset (*n* = 65). **j**, Correlation of *Cdh1* mRNA expression and age in non-small cell lung cancer (NSCLC) cell lines from c-bioportal database (*n* = 92). **k**, Correlation of *Zeb2* mRNA expression and age at diagnosis in LUAD patients from TCGA database (*n* = 491). **l-m**, Survival of *KRAS*^MUT^ LUAD patients stratified by low or high ATF4 staining score, for young (**l**) and old (**m**) patients from Swedish NSCLC-TMA cohort. Two-sided Fisher’s exact test (**a**), two-sided Chi-square test (**b,c**), simple linear regression (**d-h, j, k**), two-tailed Spearman correlation (**i**) and Log-rank test (**l,m**).

To evaluate the relevance of our findings from *KP-*Old mice in the clinical reality of patients with NSCLC, data on age, stage at diagnosis and *KRAS* mutational status were extracted from patient records for all patients diagnosed with NSCLC in Region Västra Götaland and Halland (western Sweden), between 2016 and 2018 (*n* = 997) (Fig. 5a and Extended Data Table 1). Among these, 368 (36.9%) patients harboured a mutation in *KRAS* (*KRAS*^MUT^), whereas 629 patients did not (*KRAS*^WT^). The median age at diagnosis was 71 years for the overall cohort (Extended Data Table 1) and within both mutational groups (Fig. 5b and Extended Data Table 1). We next analysed this cohort by defining two age groups: people younger than 60 years and a group of older patients spanning 65–75 years of age. The latter group corresponded to the age of *KP*-Old mice at euthanasia (±5 years) and aligned with both the median age at diagnosis of the cohort and the largest proportion, 65.2%, of all patients with *KRAS*^MUT^ diagnosed. We observed a higher prevalence of *KRAS* mutations in the 65–75-year group compared with the younger group (Fig. 5c), a finding also seen in the MSK-IMPACT^36^ dataset (*n* = 435) (Extended Data Fig. 10a). Furthermore, 65–75-year-old patients with *KRAS*^MUT^ were significantly more likely to present with loco-regionally advanced and metastatic disease (stage IIIb–IV NSCLC) compared with younger (below 60 years) patients with *KRAS*^MUT^. This pattern was not observed in patients with *KRAS*^WT^ (Fig. 5d and Extended Data Fig. 10b). Further stratification of the *KRAS*^WT^ group into subgroups based on oncogenic driver status (*EGFR* mutations, other defined drivers and unknown drivers) revealed that patients with unknown drivers showed a similar age-dependent pattern to the *KRAS*^MUT^ group. By contrast, those harbouring *EGFR* mutations or other known driver alterations showed a distinct distribution, suggesting that the observed age-associated disease progression extends beyond *KRAS* mutations, but may be specific to certain genetic subgroups (Extended Data Fig. 10b,c).

To probe tumour characteristics in relation to age, we next extracted data on primary tumour measurements from patient charts in which at least two computed tomography measurements were recorded (*n* = 464) (Fig. 5a). Among them, 172 patients harboured a mutation in *KRAS* (*KRAS*^MUT^), and 292 patients did not (*KRAS*^WT^) (Extended Data Table 2). In patients with *KRAS*^MUT^ NSCLC with loco-regionally advanced and metastatic disease (stage IIIb–IV) (*n* = 117) there was a clear decrease in primary tumour size with increasing age at diagnosis (Fig. 5e), resembling what we observe in metastasis-prone *KP*-Old mice (Fig. 1c). We did not observe a similar trend among early-stage *KRAS*^MUT^ or in *KRAS*^WT^, *EGFR*^MUT^ NSCLC and those with unknown driver mutations across all stages (Extended Data Fig. 10d–g). However, a similar age-dependent decrease in tumour size was observed in patients with ALK-positive NSCLC (Extended Data Fig. 10h). Together, these findings suggest that increased metastasis incidence despite decreased primary lung tumour burden in *KP*-Old mice reflects the clinical reality for patients with *KRAS*^MUT^ NSCLC aged 65–75 years and may extend to patients with other oncogenic drivers. Supporting this, data from many publicly available online data repositories showed a positive correlation between doubling time (hours) and age (years) in a panel of human lung cancer cell lines (*n* = 65) (Extended Data Fig. 10i), alongside upregulation of EMT markers (Extended Data Fig. 10j,k), consistent with our findings in *KP*-O cultures (Fig. 1e,f).

Our findings establish ATF4 as a key driver of ageing-induced metastasis, with its expression correlating with aggressive disease in aged *KP*-Old mice. Given the observed increase in metastatic incidence in 65–75-year-old patients with *KRAS*^MUT^, we proposed that ATF4 may serve as a biomarker for advanced disease progression. To test this, we analysed The Cancer Genome Atlas-lung adenocarcinoma cohort (TCGA-LUAD) dataset, stratifying patients based on tumour stage and ATF4 expression. Although no significant survival difference was observed in patients in the early stages (I–II) (Fig. 5f), high ATF4 expression was associated with reduced survival in patients at the advanced stages (III–IV) (median survival of 1.28 years versus 2.61 years) (Fig. 5g), reinforcing the relevance of ATF4 in human metastatic disease. Finally, to directly assess the clinical relevance of ATF4, we evaluated its expression in tumour tissues from resected patients with *KRAS*^MUT^ LUAD from a Swedish cohort^37^. In this cohort, a significant positive correlation was observed between ATF4 nuclear positivity and age at diagnosis (Fig. 5h). Age-stratified analysis confirmed significantly higher ATF4 levels in patients above the age of 65 years compared with those below 60 years (Fig. 5i). Furthermore, high ATF4 levels were associated with markedly reduced overall survival, with only 32.6% of patients with high ATF4 surviving beyond 5 years, compared with 65.7% of those with low ATF4. At 8 years, the survival disparity was even more pronounced (13.9% versus 60.0%) (Fig. 5j). The few young patients with ATF4-high tumours also have worse survival (Extended Data Fig. 10l,m). These data identify ATF4 as a robust prognostic marker of poor outcome in patients with *KRAS*^MUT^ LUAD. Collectively, these findings highlight the potential of ATF4 as a therapeutic target for mitigating ageing-driven metastatic progression in NSCLC. As older individuals represent most patients with lung adenocarcinoma, age-associated activation of ATF4 emerges as a driver of metastasis and a therapeutically actionable vulnerability in the most representative patient group.

## Discussion

Here we show that ageing is an active modifier of tumour cell state and metastatic behaviour across an aged *KRAS*-driven GEMM and several independent population-based NSCLC cohorts. Our findings place ageing as a primary driver of tumour evolutionary trajectories, which affect tumour initiation, metastasis and treatment response, and highlight the translational value of incorporating biological age into experimental and clinical study designs.

Recent studies reported that physiological ageing limits primary lung tumour growth^38,39^, seemingly at odds with age-associated increase in lung cancer mortality-to-incidence ratios^40,41^. Our work resolves this paradox by showing that ageing constrains primary tumour expansion while promoting metastatic dissemination, supporting a functional uncoupling of primary tumour growth and metastasis^42–47^. Thus, whereas metastasis may be predominantly a late event in younger individuals, it manifests earlier in older people, arguing for earlier interventions tailored for older patients diagnosed with NSCLC.

Mechanistically, age-associated chromatin states facilitate sustained ISR–ATF4 activation, driving lineage plasticity that favours dissemination over primary tumour growth. Ageing is linked to chronic cellular stress, persistent ISR activation and structural changes in the lung epithelium^48–51^. Integrating recent work identifying NUPR1-dependent iron restriction^38^ and ageing-associated proliferative restraint^39^, we position ISR–ATF4 as a central node through which ageing suppresses tumour growth while promoting ferroptosis resistance^38^, metabolic flexibility and metastatic survival.

Efficient metastatic cells prioritize dissemination over proliferation through metabolic rewiring^52–54^. Although ageing is associated with a reduced ability to induce UPR^2^, an impaired stress resolution can lead to an exaggerated and prolonged UPR response^55^. Indeed, persistent UPR–ISR signalling in aged tumour cells, supported by open chromatin at *Atf4* and reduced accessibility at UPR-resolving loci, probably sustains elevated ATF4 activity, driving glutamine-dependent metabolic plasticity and metastatic competence. This creates a selective vulnerability to glutamine-targeted interventions^53,56^. Clinically, ATF4 enrichment correlates with advanced disease, recurrence and poor survival, identifying a high-risk subgroup enriched among older patients. Notably, ATF4 expression correlates with reduced survival in advanced-stage, but not in patients with early-stage NSCLC. Furthermore, high nuclear ATF4 levels are strongly associated with disease recurrence, which is particularly enriched in older patients, thereby identifying a high-risk group that may benefit from adjuvant therapy. Younger patients with high ATF4 show outcomes comparable to older individuals, suggesting ISR–ATF4 functions as a tumour-intrinsic molecular clock^57^ reflecting physiological rather than chronological age.

Despite limitations of GEMMs in cancer research^58,59^, clinical NSCLC cohorts align with our experimental observations, showing age-related trends in tumour progression and metastasis matching our experimental models. Age-dependent progression was observed in *KRAS*-mutant and driver-unknown cases, whereas *EGFR*-driven, *BRAF*-driven, *ROS1*-driven and *RET*-driven tumours followed distinct patterns. Notably, *ALK*-driven tumours recapitulated age-associated growth suppression^38^, indicating that ageing-related effects on lung tumour progression may extend across oncogenic contexts or *TP53* status.

Future studies should explore how ageing influences treatment response and resistance, particularly in the context of immune surveillance and tumour microenvironment changes. Given the limited clinical utility of current ISR inhibitors^30^, targeting ATF4 or downstream effectors remains a promising strategy. Looking ahead, embedding age resolution in trial design and patient stratification will be key for effective clinical translation of new precision medicines for lung cancer.

## Methods

### Mice and in vivo studies

*Kras*^LSL-G12D/+^*Trp53*^flox/flox^ mice (designated as *KP*)^60^ were maintained on a mixed C57BL/6-129/Sv genetic background. Lung tumours were induced in young *KP* (2–3 months old; referred to as *KP*-Young) and old *KP* (18–19 months old, referred to as *KP*-Old) mice through intratracheal instillation with Lenti-Cre as described in ref. ^61^ or with Ad5mSPC-Cre viral particles (PFU 2 × 10^7^; University of Iowa; VVC-Berns-1168) under general anaesthesia as described in ref. ^10^.

*NXG* (NOD Xenograft Gamma) mice (*NOD-Prkdc*^scid^-*IL2rg*^Tm1^/Rj) were obtained from Janvier Laboratories and were 6–10 weeks old at the start of the experiments. All transplantation studies were conducted using age-matched cohorts of mice. For subcutaneous implantations, a total of 2.5 × 10^5^ of green fluorescent protein (GFP)-luciferase-expressing versions of the indicated cells suspended in PBS were subcutaneously injected into the lower right and lower left flanks of *NXG* mice. Tumours were measured every other day by callipers and the tumour volume was calculated using the formula volume = (length × width^2^)/2. At no point during the study did any tumour exceed the permitted maximal measurements of 2 cm × 2 cm or endpoint criteria.

For intravenous injections, a total of 5 × 10^4^ of the indicated cells were injected into the lateral tail vein of *NXG* mice. All transplantation experiments were performed in young immunodeficient *NXG* hosts to minimize confounding by host age or immunity.

For CB-839 studies, mice were randomized and subjected to treatment with either 200 mg kg^−1^ body weight CB-839 (no. HY-12248, MedChemExpress) or vehicle. Treatments were administered twice a day every other day following either the tumour-establishment phase (subcutaneous implantations) or within 6 h after the bloodstream injection of cells. The vehicle control consisted of 25% (w/v) 2-hydroxypropyl-β-cyclodextrin in 10 mM citrate buffer (pH 2.0).

For doxycycline-induced *Atf4* silencing, 1 mg ml^−1^ of doxycycline (no. HY-N0565B, MedChemExpress) was administered in the drinking water supplemented with 5% sucrose. For ISRIB (no. HY-12495, MedChemExpress) studies, indicated cells were pretreated 3 times a day for 7 days with either ISRIB (2 µM) or vehicle (dimethylsulfoxide, DMSO) and then injected into mice. ISRIB was administered at 10 mg kg^−1^ twice daily by intraperitoneal injection or vehicle alone (45% saline, 50% PEG 400, 5% DMSO).

Same-sex mice were housed in individually ventilated cages, under a 12–12 h light–dark cycle, with ambient temperature (15–21 °C) and humidity control (45–70% relative humidity), enrichment material and ab libitum rodent chow and water. All mouse experiments described in this study were approved by the Research Animal Ethics Committee in Gothenburg (2071/19; 2077/19 and 6057/24).

### IVIS imaging

Mice were injected intraperitoneally with 30 mg ml^−1^ of d-luciferin (no. 15225733, Fisher Scientific) and organs were imaged ex vivo 10 min after injection. Luminescence was quantified as constant, or as total flux (p s^−1^). Analysis was performed with Living Image v.4.7.4 software, maintaining the region of interest over the tissues at a constant size. The radiance (p s^−1^ cm^−2^ sr^−1^) from the region of interest was normalized against the background radiance.

### Histology and IHC analyses

Lungs were perfused through the trachea with PBS, fixed overnight, transferred to 70% ethanol, embedded in paraffin, cut into 5-µm sections and stained with haematoxylin and eosin (H&E). Slides were scanned using Olympus Slideview VS200. Tumour burden (percentage tumour area per lung area) in H&E-stained sections of all five lung lobes was quantified using BioPix iQ software (v.2.1.4). Histological tumour grading was done on non-necrotic lesions according to a previously described protocol in ref. ^10^. For IHC analyses of mouse and human lung sections as well as tissue microarray (TMA) sections from a Swedish NSCLC cohort^37^, deparaffinization was followed by epitope retrieval and blocking of endogenous peroxidase activity with H_2_O_2_. Sections were then incubated with primary antibodies listed in Supplementary Table 4. Slides were scanned at ×40 on a Hamamatzu Nanozoomer (2.0HT). The total number of positively stained cells in tumours was counted and normalized to either the tumour area or the total number of cells.

### Cells

Primary tumour cultures were isolated from lung tumours of *KP*-Young and *KP*-Old mice roughly 30 weeks following tumour induction. Primary tumour cultures referred to as *KP*-Y and *KP-*O were isolated with the tumour dissociation kit for mouse (no. 130-096-730, MACS Miltenyi Biotec) and the gentleMACS Octo Dissociator (no. 130-093-235, MACS Miltenyi Biotec). A549 cell line, a human lung adenocarcinoma epithelial cell line, was obtained from the American Type Culture Collection (catalogue no. CCL-185). Primary cultures as well as the cells were maintained in DMEM medium (Fisher Scientific) supplemented with 0.1% gentamycin (10 mg ml^−1^, Fisher Scientific), 2% l-glutamine (200 mM, Fisher Scientific) and 10% fetal bovine serum (Thermo Fisher). All cell lines were routinely tested negative for mycoplasma. For the different assays, cells were seeded in Roswell Park Memorial Institute (RPMI) media (Fisher Scientific) supplemented with 10% fetal bovine serum; 1% l-glutamine and 0.1% gentamicin.

### Proliferation and viability assays

For population doublings assays, *KP*-Y1, *KP*-Y2 and *KP*-O1, *KP*-O2 cells were seeded in triplicate in 6-well plates, counted 3 days later and reseeded at the same initial density, for a total of 12–15 days. For cell viability assays, cells were plated in a white, opaque 96-well plate with clear bottom at a density of 2.5 × 10^3^ cells per well in RPMI. 24 h after seeding, drugs were added at the indicated concentrations. Then 72 h after drug addition, cell viability in the presence of all the compounds was assessed by CellTiter-Glo 2.0 (no. G9242, Promega) with a spectrophotometer (Synergy BioTex HTX).

### Clonogenic assay

A total of 5,000 *KP*-Y1, *KP*-Y2 and *KP*-O1, *KP*-O2 cells were seeded in triplicates in 10 cm plates in RPMI media supplemented with 2 mM, 1 mM, 0.5 mM or 0.25 mM concentrations of l-glutamine for 6 days. For clonogenic assays with ISRIB, *KP*-Y1 and *KP*-O1 cells were treated with 2 µM ISRIB 3 times a day for 7 days, and a total of 1,000 *KP*-Y1 and *KP*-O1 cells were seeded in triplicates in 10-cm plates in DMEM media supplemented with 2 µM ISRIB or vehicle (DMSO) for another 7 days. Colonies were fixed and stained by incubation in PBS containing 0.05% crystal violet, 1% formaldehyde and 1% methanol for 20 min. The number of colonies was counted with OpenCFU^62^.

### 3D cultures and anoikis resistance assay

Here 20 × 10^3^ cells were seeded in an ultra-low attachment plate (no. 10023683, Corning) and in parallel in a normal attachment 96-well plate in 3–6 replicates per cell line. For ISRIB experiments, indicated cells were pretreated 3 times a day for 7 days with either ISRIB (2 µM) or vehicle (DMSO) before seeding into ultra-low-attachment plates. After 48 h of cell seeding, cell viability was assessed by CellTiter-Glo 2.0 (no. G9242, Promega) with spectrophotometer (Synergy BioTex HTX). Cell viability values from ultra-low-attachment conditions were normalized to those from adherent plates that was quantified 16 h after seeding.

### Spheroid formation and collagen invasion assays

Here 20 × 10^3^ cells were seeded in an ultra-low attachment plate (no. 10023683, Corning) with 10% Matrigel (no. 11523550, Fisher Scientific). For collagen invasion assays, 10 × 10^3^ cells were seeded in an ultra-low attachment plate (no. 10023683, Corning). The next day, cells were embedded in collagen (no. ECM675, Sigma-Aldrich). Images were acquired using Zeiss Axio Observer.A1 reverse microscope with an AxioCam MRm camera (Carl Zeiss) 24–48 h later.

### Caspase activity assay

Briefly, 20 × 10^3^ cells were seeded in an ultra-low attachment plate (no. 10023683, Corning) and in parallel in a normal attachment 96-well plate in 5 replicates per cell line. After 48 h of cell seeding, caspase activity in cells was measured using the commercially available Caspase-Glo 3/7 Assay (no. G8090, Promega). Values from ultra-low-attachment conditions were normalized to those from adherent plates that was quantified 16 h after seeding.

### Cell trace proliferation assay

Cells were labelled using the CellTrace Proliferation kit (no. C34554, Thermo Fisher Scientific) according to the manufacturer’s instructions and seeded in low attachment plates to form spheroids. Single-cell suspensions were prepared from spheroids on days 0, 2, 4, 6 and 8, flow cytometry data were collected on Sony ID7000 Spectral Cell Analyzer and analysed using Flow Jo software v.10.8.1.

### Lentiviral production and transduction

Lentiviruses were produced by transfecting 293FT packaging cells (no. R70007, Life Technologies) with lentiviral backbone constructs, packaging plasmid psPAX2 (Addgene plasmid no. 12260) and envelope plasmid pMD2.G (Addgene plasmid no. 12259) using the X-tremeGENE 9 DNA Transfection Reagent (no. 6365809001, Sigma-Aldrich). Lentiviral supernatants were collected 2 days after transfection. Target cells were transduced once with lentiviruses supplemented with 8 μg ml^−1^ polybrene (no. TR-1003-G, Sigma-Aldrich) and selected with puromycin (2 μg ml^−1^, no. 12122530, Fisher Scientific). Luciferase-GFP-expressing vector pLV[Exp]-EGFP:T2A: Hygro-EF1A>Luc2 was generated by VectorBuilder. The GFP expression was validated using flow cytometry. For CRISPR-mediated gene knockout, the LentiCRISPRv2 (Addgene plasmid no. 52961) vector was digested with BsmBI and ligated with BsmBI-compatible pre-annealed oligonucleotides^63,64^. For overexpression of ATF4 in primary cultures as well as A549, plasmids TFORF3549 and TFORF3036 were a gift from F. Zhang (Addgene plasmids nos. 145025 and 144512)^65^. The expression of target proteins in CRISPR-knockout and overexpression experiments were evaluated by western blotting 3–5 days after selection. Sequences are provided in Supplementary Table 3.

### shRNA-mediated knockdown

Doxycycline-induced knockdown of *Atf4* was achieved by cloning miR-E shRNAs targeting *Atf4* into the LT3GEPIR vector as previously described in detail in ref. ^66^. In brief, LT3GEPIR was digested with XhoI and EcoRI, and purified with a gel extraction kit (no. 28704, Qiagen). Single-stranded ultramers were amplified with forward primer miRE-XhoI and reverse primer mirE-EcoRI. Amplicons were gel-purified, digested with XhoI and EcoRI, cleaned up with a PCR purification kit (no. 28104, Qiagen) and ligated into the cut LT3GEPIR vector with T4 DNA Ligase at a 3:1 insert:vector molar ratio. Vectors were transduced into cells and selected with 2 µg ml^−1^ puromycin for 2 days. Knockdown of ATF4 was verified by western blot analysis following 72 h of treatment with 1 µg ml^−1^ doxycycline (no. D9891, Sigma-Aldrich). All experiments performed with shRNA-mediated knockdown were performed following 72 h treatment with 1 µg ml^−1^ doxycycline. Sequences are provided in Supplementary Table 3.

### Western blotting

Proteins were isolated by using the 2× Laemmli Buffer (no. 1610737, BioRad) supplemented with β-mercaptoethanol (no. M3148, Sigma-Aldrich). Samples were subsequently heated at 95 °C for 10 min. Proteins were separated on 4–20% Mini-PROTEAN TGX Stain-Free gel (no. 4568096, BioRad) and then transferred onto a 0.2 µM nitrocellulose membrane (BioRad), incubated with specific primary antibodies listed in Supplementary Table 4. Protein bands were detected using Clarity Western ECL substrate (no. 1705061, BioRad) with the Amersham ImageQuant 800 western blot imaging systems (cytiva). A list of antibodies used is provided in Supplementary Table 4.

### Real-time quantitative PCR

RNA was isolated with the RNeasy Plus Mini kit (no. 74136, Qiagen), and complementary DNA (cDNA) was synthesized using iScript Adv cDNA Kit for RT–qPCR (no. 1725038, BioRad). Gene expression was analysed using the PowerUp SYBR Green Master Mix (no. A25777, Thermo Fisher Scientific) on QuantStudio 5 Real-Time PCR system (Thermo Fisher). The list of primers used is provided in Supplementary Table 3.

### Reagents

Cells attached or in spheroids were treated with indicated drugs at the following concentrations, unless stated otherwise in the figure: vehicle (DMSO D8418, Sigma-Aldrich), 100 nM CB-839 (5337170001, Sigma-Aldrich), 2 mM pyruvate (11501871; Fisher Scientific); 50 µM Trolox (648471, Sigma-Aldrich); 2 mM DMG (349631, Sigma-Aldrich), 500 µM *N*-acetyl-l-cysteine (A7250, Sigma-Aldrich), 0.3 µM erastin (E7781, Sigma-Aldrich); 20 µM 5-methyltetrahydrofolic acid disodium salt (M0132, Sigma-Aldrich); 5 µM hypoxanthine (H9636, Sigma-Aldrich), 1× nucleosides (ES-008-D, Sigma-Aldrich), 1× non-essential amino acids (SH30238.01, Nordic Biolabs), 2 mM l-serine (S4311, Sigma-Aldrich), 2 mM l-asparagine (A4159, Sigma-Aldrich), 2 µM folic acid (F8758, Sigma-Aldrich), 50 µM thymidine (T1895, Sigma-Aldrich), 1 mM sodium formate (247596, Sigma-Aldrich), 20 µM l-aspartic acid (A7219, Sigma-Aldrich), 2 µM ISRIB (HY-12495, MedChemExpress), bis-2-(5-phenylacetamido-1,3,4-thiadiazol-2-yl) ethyl sulfide (BPTES, HY-12683, MedChemExpress), V-9302 (HY-112683, MedChemExpress), ERG240 (HY-W193545A, MedChemExpress), branched-chain aminotransferase inhibitor (HY-116044, MedChemExpress), NCT-503 (HY-101966, MedChemExpress), l-6-diazo-5-oxonorleucine (DON, HY-108357, MedChemExpress), 2-DG (Sigma-Aldrich), 10 µM MG-132 (HY-13259; MedChemExpress), 50 ng ml^−1^ actinomycin D (SBR00013-1ML; Sigma-Aldrich), 20 µg ml^−1^ cycloheximide solution (C4859, Sigma-Aldrich), 5 µM or 1 µM of PERK inhibitor (GSK2656157, HY 13820, MedChemExpress).

### Mitochondrial respiration

Oxygen consumption rate (OCR) and extracellular acidification rate experiments were performed using the XF96pro apparatus from Seahorse Bioscience (Agilent). Cells were seeded to roughly 90% confluence for each condition in RPMI. The following day, media was completely replaced with RPMI pH 7.4 (103576-100, Agilent) containing 10 mM glucose and 2 mM glutamine and incubated for 45 min at 37 °C in a CO_2_-free incubator before measurements. Basal and maximal respiration measurements were obtained by performing a mito-stress test (103015-100, Agilent) following the manufacturer’s instructions. Data were normalized to protein content using Pierce BCA Protein Assay Kit (23225, ThermoScientific).

### GC–MS analysis of polar metabolites and stable isotope tracing

For analysis of tumour primary cultures in attached conditions, 2.5 × 10^5^ cells were seeded in 6-well plates containing 2 ml of stable isotope RPMI media supplemented with 10% dialysed fetal bovine serum with 2 mM [U13C]-l-glutamine (Cambridge Isotope Laboratories), 10 mM [1,2-13C]-d-glucose (Sigma-Aldrich) or [3-13 C]-d-glucose (Sigma-Aldrich) for 8 h. Cells were washed twice in ice-cold saline and then collected by scraping in 200 µl of 80% (v/v) ice-cold methanol containing 1 µg ml^−1^ norvaline (Sigma-Aldrich). Samples were then vortexed for 10 min at 4 °C and then centrifuged at maximum speed for 10 min. The supernatant was transferred to fresh tubes and then dried in a speed vac. Dried metabolite extracts were then derivatized with 20 ml of *O*-methoxyamine-hydrochloride reagent (89803, Sigma-Aldrich) in pyridine (270407, Sigma-Aldrich) at a concentration of 20 mg ml^−1^ for 60 min at 37 °C and 20 ml of *N*-*tert*-butyldimethylsilyl-*N*-methyltrifluoracetamide with 1% *tert*-butyldimethylchlorosilane (375934, Sigma-Aldrich) for 60 min at 37 °C. After derivatization, samples were analysed by gas chromatography with mass spectrometry (GC–MS) using a DB-35ms column (Agilent Technologies) in an Agilent Intuvo gas chromatograph coupled to an Agilent 5977B mass spectrometer. Helium was used as the carrier gas at a flow rate of 1.2 ml min^−1^. Then 1 µl of sample was injected in split mode (split 1:1) at 270 °C. After injection, the GC oven was held at 100 °C for 1 min and then increased to 300 °C at 3.5 °C min^−1^. The oven was then ramped to 320 °C at 20 °C min^−1^ and held for 5 min at 320 °C. The MS system operated under electron impact ionization at 70 eV and the MS source and quadrupole were held at 230 °C and 150 °C, respectively, the detector was used in scanning mode, and the scanned ion range was 10–650 *m/z*. Mass isotopomer distributions were determined by integrating the appropriate ion fragments for each metabolite^67^ using MATLAB (Mathworks) and an algorithm adapted from ref. ^68^ that corrects for natural abundance. For all data, total or relative metabolite pool sizes are normalized to cell counts for each condition.

### RNA-seq

Total RNAs were extracted with the RNeasy Plus Mini kit (74136, Qiagen). RNA quality was assessed using the DNF-471 RNA kit on a fragment analyser (Agilent). RNA-seq libraries were prepared according to the Smart-seq2 protocol, developed^69^ with some minor modifications. Reverse transcription followed by preamplification of purified cDNA was performed as described in ref. ^70^. Samples were purified using Agencourt AMPure XP beads (BD Bioscience). Purified samples were further used for library preparation with the Nextera XT DNA library preparation kit and Nextera XT index kit v2 (Illumina), according to the manufacturer’s recommendations. Samples were indexed and amplified by adding 15 µl of Nextera PCR master mix and 5 µl of each index, i7 and i5, obtaining a reaction volume of 50 µl. Amplification and indexing were performed in a T100 instrument at 95 °C for 10 s, 55 °C for 30 s, and as a final step an incubation at 72 °C for 5 min.

Using Agencourt AMPure XP (BD Bioscience) samples were again purified by following the manufacturer’s protocol. Concentrations were measured using Qubit double-stranded DNA high sensitivity Assay Kit, on a Qubit 4 fluorometer (Invitrogen, Thermo Fisher Scientific). The quality control and the fragment size distribution were determined using the DNF-474 High sensitivity next-generation sequencing kit on a Fragment analyser (Agilent).

Libraries were pooled and sequencing was performed at the Genomics core facility at the University of Gothenburg on a NextSeq 500 instrument (Illumina) using the NextSeq 500/500 High Output Kit v.2.5 (150 cycles) and paired-end sequencing (2 × 75 cycles).

Indexing of the ENSEMBL GRCm39 reference genome as well as alignment of sequencing reads was performed using STAR version 2.7.9a (ref. ^71^). Read count of aligned reads was performed with HTSeq version 0.9.1 (ref. ^72^). Before differential expression analysis, genes with an average read count of less than 3 were excluded. Differential expression analysis was performed with DESeq2 version 1.34.0 (ref. ^73^) in R version 4.1.2. First, the four replicate samples within each cell line were merged using the collapseReplicates function. Subsequently, the analysis was run comparing the two groups of primary cultures, Young and Old as well as between *KP*-Y o/e and *KP*-Y control. Significantly regulated genes were defined as having at least twofold regulation with an adjusted *P* value (Benjamini–Hochberg) less than or equal to 0.05. GSEA was done using the GSEA desktop v.4.3.3 (ref. ^74^). Functional annotation of genes by Gene Ontology and pathway analysis by Kyoto Encyclopedia of Genes and Genomes were done using DAVID^75^. Protein–protein association data was obtained from the STRING database (v.12.0) and integrated to analyse both functional and physical interactions using curated datasets and high-throughput experimental evidence. Computational predictions and experimental data were combined to construct and score protein networks, which was subsequently analysed for enriched biological functions and potential input biases.

### ATAC-seq

Library construction for ATAC-seq of paired-end 150-bp-long reads by the Illumina HiSeq was performed at GENEWIZ Azenta Life Sciences. In brief, sequencing adaptors and low-quality bases were trimmed using Trimmomatic v.0.38. Cleaned reads were next aligned to reference genome mm10 using Bowtie2. Aligned reads were filtered using samtools v.1.9 to keep alignments that have a minimum mapping quality of 30. PCR or optical duplicates were marked using Picard v.2.18.26 and removed. Before peak calling, reads mapping to mitochondria (mt) were called and filtered, and reads mapping to unplaced contigs were removed. MACS2 v.2.1.2 was used for peak calling to identify open chromatin regions. Called peaks were filtered for blacklisted regions to mitigate errors due to mappability. Valid peaks from each group were merged and peaks called in at least 66% of samples are kept for downstream analyses. Reads falling beneath peaks were counted in all samples, and these counts were used for differential peak analyses using the R package Diffbind (v.3.20.0). All sequencing tracks, bigWig files were viewed using the Integrated Genomic Viewer (v.2.16.2). GREAT analysis was done to identify the enriched pathways from ATAC-seq.

### Western Sweden patient cohort

All patients in western Sweden (eight hospitals) diagnosed with NSCLC from the years 2016 to 2018 and molecularly assessed were included (*n* = 997). Age at diagnosis, histology and tumour size at diagnosis from computed tomography scans were obtained from patient charts. Patients were assessed with next-generation sequencing for mutational status on DNA from formalin-fixed paraffin-embedded blocks or cytological smears using the Ion AmpliSeq Colon and Lung Cancer Panel v2 from Thermo Fisher Scientific as a part of the diagnostic workup process at the Department of Clinical Pathology at Sahlgrenska University Hospital, assessing hotspot mutations in *EGFR*, *BRAF*, *KRAS* and *NRAS*.

To obtain the most recent and accurate untreated primary tumour size, measurements were collected from the radiology report of computed tomography performed before a final diagnosis of NSCLC was established. For patients with two or three tumour dimensions available in their charts, tumour volume was calculated using mathematical formulas^76^. The formula used for tumours with two dimensions is volume = (width × width × length/2), where width is the lowest measurement and length is the highest measurement. For tumours with three dimensions, the tumour volume was calculated as follows: volume = width × length × height. Tumour volume sizes were converted to the same unit of cm^3^. Pearson correlation was used to identify the linear correlation between tumour volume and age for patients with *KRAS* mutated and with *KRAS* wild type. Approval from the Swedish Ethical Review Authority (Dnr 2019-04771) was obtained before the commencement of the study. No informed consent was required because all data were presented in a de-identified form according to the Swedish Ethical Review Authority.

### TMA cohort

TMAs from the Swedish NSCLC cohort included primary NSCLC resections (1995–2005)^37^. All procedures complied with Swedish legislation and were approved by the Uppsala Ethical Review Board (2006/325). All KRAS-mutant lung adenocarcinomas were selected; ATF4 expression was scored and correlation with survival outcomes analysed.

### Statistics and reproducibility

GraphPad Prism (v.9.0 and v.10.0) software was used for statistical analyses. Data are presented as mean ± standard error of the mean (s.e.m.), unless otherwise specified. All statistical tests were two-tailed, and replicates represent biological replicates unless otherwise stated. *P* values were calculated using two-sided Student’s *t*-tests for measurements of tumour burden or for comparisons between two groups, unless indicated otherwise in the figure legends. For contingency tables, Fisher’s exact test or the chi-squared test was used, as appropriate. One-way analysis of variance (ANOVA) with Tukey’s post hoc test was used for comparisons among several groups, and two-way ANOVA was used for analyses involving several groups across time. Statistical tests used for each experiment are indicated in the corresponding figure legends. A *P* value less than 0.05 was considered statistically significant. Western blot experiments were replicated at least three times with reproducible results. When representative images are shown, a minimum of three samples per group from the larger cohort were evaluated. All in vitro assays were performed at least three times with a minimum of *n* = 3 biological replicates per group. For in vivo experiments, the minimum sample size was three independent mice or tumours, with exact sample sizes per condition reported in the figure legends. Spearman’s *r* was calculated to analyse the correlation of age with EMT marker expression. Patients were divided into two groups according to age at diagnosis and compared for *KRAS* gene alteration frequencies. No statistical methods were used to predetermine sample size. Individual data points are shown as one dot per sample. Samples and experimental animals were randomly assigned to experimental groups, and sample collection was performed randomly when possible.

### Reporting summary

Further information on research design is available in the Nature Portfolio Reporting Summary linked to this article.

## Online content

Any methods, additional references, Nature Portfolio reporting summaries, source data, extended data, supplementary information, acknowledgements, peer review information; details of author contributions and competing interests; and statements of data and code availability are available at 10.1038/s41586-026-10216-0.

## Supplementary information

Supplementary Fig. 1 Gel source data. All uncropped western blot images with protein ladder markers.

Reporting Summary

Supplementary Table 1 Gene lists related to Figs. 2 and 3.

Supplementary Table 2 Lists related to Extended Data Figs. 4 and 5.

Supplementary Table 3 Oligonucleotide sequences used in the study.

Supplementary Table 4 Antibodies used in the study.

## Source data

Source Data Fig. 1

Source Data Fig. 2

Source Data Fig. 3

Source Data Fig. 4

Source Data Extended Data Fig. 1

Source Data Extended Data Fig. 6

Source Data Extended Data Fig. 9

## Figures and Tables

**Extended Data Table 1:**
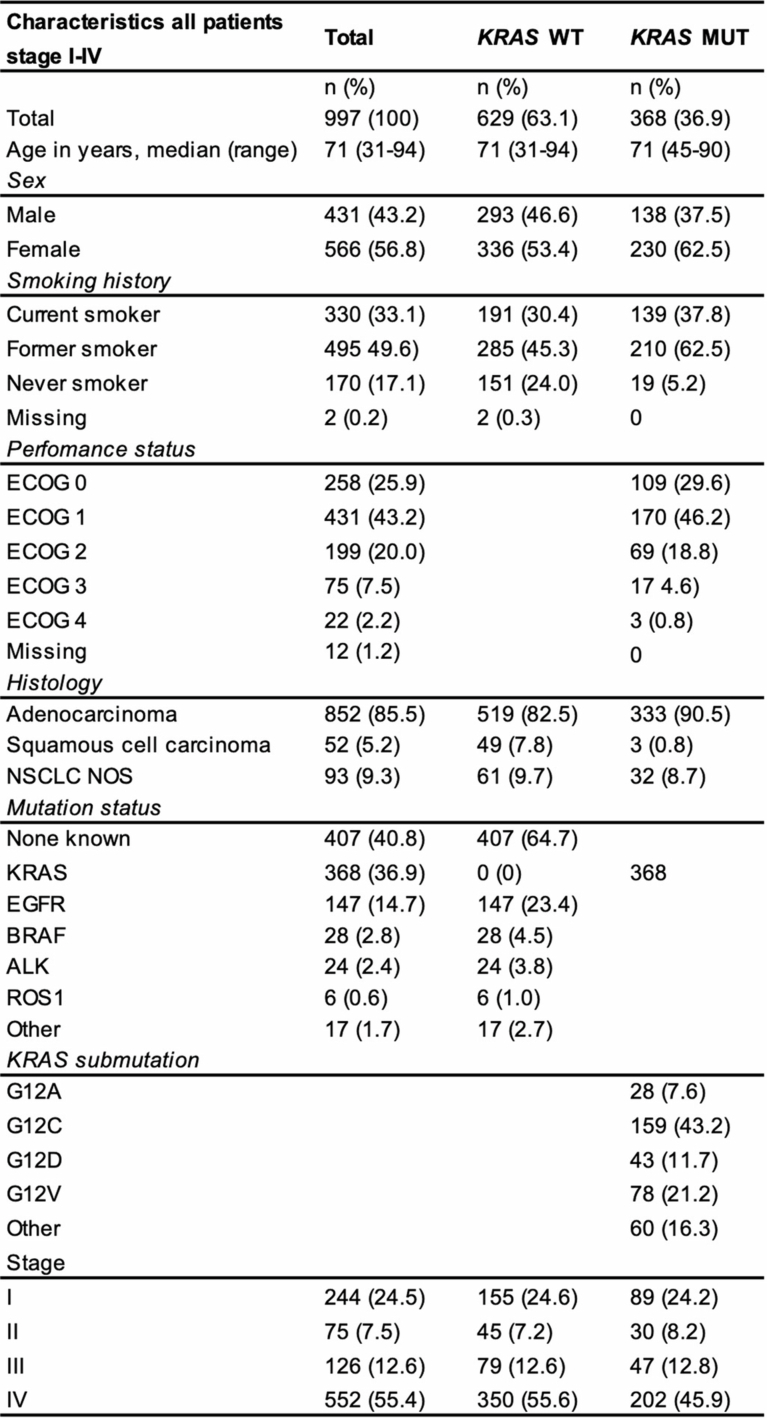
Baseline characteristics of patients included in the western Sweden cohort (2016–2018)

**Extended Data Table 2:**
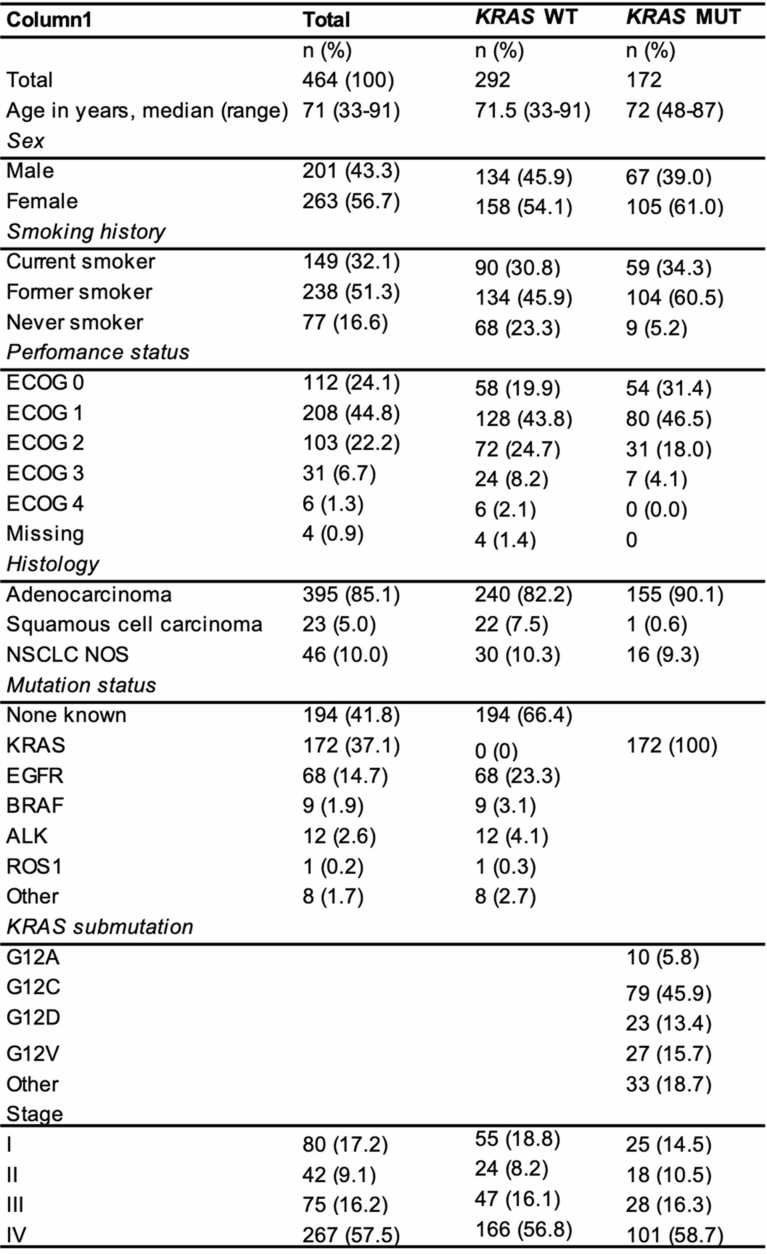
Characteristics of patients having their primary tumour volume calculated

## Data Availability

RNA-seq raw and associated processed data from primary *KP*-Y and *KP*-O cultures with or without ATF4 overexpression and RNA-seq, ATAC-seq raw and associated processed data from *KP*-Y and *KP*-O primary cultures have been deposited in the Gene Expression Omnibus under accession codes GSE263689. All the other data and raw gel images are included with the paper. All the databases used in this study are publicly available. Gene expression profiles and clinical information for patients with lung adenocarcinoma were obtained from the TCGA-LUAD available at https://www.cancer.gov/tcga. Genomic alteration and clinical data were accessed through the cBioPortal for Cancer Genomics available at https://www.cbioportal.org. Information regarding lung adenocarcinoma organotropism was obtained from MSK-IMPACT data^36^. Gene expression and dependency data for cancer cell lines were obtained from the Cancer Cell Line Encyclopedia and DepMap portal (version 1, Public 23Q2) available at https://depmap.org/portal (ref. ^77^). Population doubling times and cell line age annotations were retrieved from the Cellosaurus database at https://www.cellosaurus.org/. Protein–protein interaction analyses were performed using the STRING database available at https://string-db.org/. Source data are provided with this paper.

## References

[1] Siegel, R. L., Giaquinto, A. N. & Jemal, A. Cancer statistics, 2024. *CA Cancer J. Clin.* **74**, 12–49 (2024). 38230766 10.3322/caac.21820

[2] López-Otín, C., Blasco, M. A., Partridge, L., Serrano, M. & Kroemer, G. The hallmarks of aging. *Cell* **153**, 1194–1217 (2013). 23746838 10.1016/j.cell.2013.05.039 PMC3836174

[3] Mani, K. et al. Causes of death among people living with metastatic cancer. *Nat. Commun.* **15**, 1519 (2024). 38374318 10.1038/s41467-024-45307-x PMC10876661

[4] Ganesh, K. & Massagué, J. Targeting metastatic cancer. *Nat. Med.* **27**, 34–44 (2021). 33442008 10.1038/s41591-020-01195-4 PMC7895475

[5] Surveillance, Epidemiology, and End Results Program (SEER). Cancer Stat Facts: Lung and Bronchus Cancer https://seer.cancer.gov/statfacts/html/lungb.html (National Cancer Institute, 2023).

[6] Lazure, F. & Gomes, A. P. Cancer progression through the lens of age-induced metabolic reprogramming. *Nat. Rev. Cancer* **25**, 801–817 (2025). 40646271 10.1038/s41568-025-00845-4

[7] Yang, W. H., Qiu, Y., Stamatatos, O., Janowitz, T. & Lukey, M. J. Enhancing the efficacy of glutamine metabolism inhibitors in cancer therapy. *Trends Cancer* **7**, 790–804 (2021). 34020912 10.1016/j.trecan.2021.04.003 PMC9064286

[8] Vasan, K. & Chandel, N. S. Molecular and cellular mechanisms underlying the failure of mitochondrial metabolism drugs in cancer clinical trials. *J. Clin. Invest.* **134**, e176736 (2024). 38299592 10.1172/JCI176736 PMC10836798

[9] Yuan, R., Peters, L. L. & Paigen, B. Mice as a mammalian model for research on the genetics of aging. *ILAR J.* **52**, 4–15 (2011). 21411853 10.1093/ilar.52.1.4 PMC3074346

[10] DuPage, M., Dooley, A. L. & Jacks, T. Conditional mouse lung cancer models using adenoviral or lentiviral delivery of Cre recombinase. *Nat. Protoc.* **4**, 1064–1072 (2009). 19561589 10.1038/nprot.2009.95 PMC2757265

[11] Ecker, B. L. et al. Age-related changes in HAPLN1 Increase lymphatic permeability and affect routes of melanoma metastasis. *Cancer Discov.* **9**, 82–95 (2019). 30279172 10.1158/2159-8290.CD-18-0168 PMC6328344

[12] Kaur, A. et al. Remodeling of the collagen matrix in aging skin promotes melanoma metastasis and affects immune cell motility. *Cancer Discov.* **9**, 64–81 (2019). 30279173 10.1158/2159-8290.CD-18-0193 PMC6328333

[13] Flurkey, K. & Currer, J. M. Pitfalls of animal model systems in ageing research. *Best Pract. Res. Clin. Endocrinol. Metab.* **18**, 407–421 (2004). 15261846 10.1016/j.beem.2004.02.001

[14] Henry, C. J. & DeGregori, J. Modelling the ageing dependence of cancer evolutionary trajectories. *Nat. Rev. Cancer* **25**, 757–780 (2025). 40640377 10.1038/s41568-025-00838-3

[15] Winslow, M. M. et al. Suppression of lung adenocarcinoma progression by Nkx2-1. *Nature* **473**, 101–104 (2011). 21471965 10.1038/nature09881 PMC3088778

[16] Sutherland, K. D. et al. Multiple cells-of-origin of mutant K-Ras-induced mouse lung adenocarcinoma. *Proc. Natl Acad. Sci. USA* **111**, 4952–4957 (2014). 24586047 10.1073/pnas.1319963111 PMC3977239

[17] Pagiatakis, C., Musolino, E., Gornati, R., Bernardini, G. & Papait, R. Epigenetics of aging and disease: a brief overview. *Aging Clin. Exp. Res.* **33**, 737–745 (2021). 31811572 10.1007/s40520-019-01430-0 PMC8084772

[18] Kabacik, S. et al. The relationship between epigenetic age and the hallmarks of aging in human cells. *Nat. Aging* **2**, 484–493 (2022). 37034474 10.1038/s43587-022-00220-0 PMC10077971

[19] Wang, K. et al. Epigenetic regulation of aging: implications for interventions of aging and diseases. *Signal Transduct. Targ. Ther.* **7**, 374 (2022). 10.1038/s41392-022-01211-8 PMC9637765 36336680

[20] Yang, J. H. et al. Loss of epigenetic information as a cause of mammalian aging. *Cell* **186**, 305–326 (2023). 36638792 10.1016/j.cell.2022.12.027 PMC10166133

[21] Pakos-Zebrucka, K. et al. The integrated stress response. *EMBO Rep.* **17**, 1374–1395 (2016). 27629041 10.15252/embr.201642195 PMC5048378

[22] Gomez, J. A. & Rutkowski, D. T. Experimental reconstitution of chronic ER stress in the liver reveals feedback suppression of BiP mRNA expression. *eLife* **5**, e20390 (2016). 27938665 10.7554/eLife.20390 PMC5179193

[23] Fonseca, S. G. et al. Wolfram syndrome 1 gene negatively regulates ER stress signaling in rodent and human cells. *J. Clin. Invest.* **120**, 744–755 (2010). 20160352 10.1172/JCI39678 PMC2827948

[24] Novoa, I., Zeng, H., Harding, H. P. & Ron, D. Feedback inhibition of the unfolded protein response by GADD34-mediated dephosphorylation of eIF2alpha. *J. Cell Biol.* **153**, 1011–1022 (2001). 11381086 10.1083/jcb.153.5.1011 PMC2174339

[25] Huang, C. H., Chu, Y. R., Ye, Y. & Chen, X. Role of HERP and a HERP-related protein in HRD1-dependent protein degradation at the endoplasmic reticulum. *J. Biol. Chem.* **289**, 4444–4454 (2014). 24366871 10.1074/jbc.M113.519561 PMC3924306

[26] Ali, D. M., Ansari, S. S., Zepp, M., Knapp-Mohammady, M. & Berger, M. R. Optineurin downregulation induces endoplasmic reticulum stress, chaperone-mediated autophagy, and apoptosis in pancreatic cancer cells. *Cell Death Discov.* **5**, 128 (2019). 31428460 10.1038/s41420-019-0206-2 PMC6689035

[27] Pällmann, N. et al. Regulation of the unfolded protein response through ATF4 and FAM129A in prostate cancer. *Oncogene* **38**, 6301–6318 (2019). 31312022 10.1038/s41388-019-0879-2

[28] Verginadis, I. I. et al. A stromal integrated stress response activates perivascular cancer-associated fibroblasts to drive angiogenesis and tumour progression. *Nat. Cell Biol.* **24**, 940–953 (2022). 35654839 10.1038/s41556-022-00918-8 PMC9203279

[29] Dey, S. et al. ATF4-dependent induction of heme oxygenase 1 prevents anoikis and promotes metastasis. *J. Clin. Invest.* **125**, 2592–2608 (2015). 26011642 10.1172/JCI78031 PMC4563676

[30] Nguyen, H. G. et al. Development of a stress response therapy targeting aggressive prostate cancer. *Sci. Transl. Med.* **10**, eaar2036 (2018). 29720449 10.1126/scitranslmed.aar2036 PMC6045425

[31] Payen, V. L., Porporato, P. E., Baselet, B. & Sonveaux, P. Metabolic changes associated with tumor metastasis, part 1: tumor pH, glycolysis and the pentose phosphate pathway. *Cell. Mol. Life Sci.* **73**, 1333–1348 (2016). 26626411 10.1007/s00018-015-2098-5 PMC11108399

[32] Aurora, A. B. et al. Loss of glucose 6-phosphate dehydrogenase function increases oxidative stress and glutaminolysis in metastasizing melanoma cells. *Proc. Natl Acad. Sci. USA* **119**, e2120617119 (2022). 35110412 10.1073/pnas.2120617119 PMC8833200

[33] Fendt, S. M., Frezza, C. & Erez, A. Targeting metabolic plasticity and flexibility dynamics for cancer therapy. *Cancer Discov.* **10**, 1797–1807 (2020). 33139243 10.1158/2159-8290.CD-20-0844 PMC7710573

[34] Sayin, V. I. et al. Activation of the NRF2 antioxidant program generates an imbalance in central carbon metabolism in cancer. *eLife* **6**, e28083 (2017). 28967864 10.7554/eLife.28083 PMC5624783

[35] Muir, A. et al. Environmental cystine drives glutamine anaplerosis and sensitizes cancer cells to glutaminase inhibition. *eLife* **6**, e27713 (2017). 28826492 10.7554/eLife.27713 PMC5589418

[36] Lengel, H. B. et al. Genomic mapping of metastatic organotropism in lung adenocarcinoma. *Cancer Cell* **41**, 970–985 (2023). 37084736 10.1016/j.ccell.2023.03.018 PMC10391526

[37] Botling, J. et al. Biomarker discovery in non–small cell lung cancer: integrating gene expression profiling, meta-analysis, and tissue microarray validation. *Clin. Cancer Res.* **19**, 194–204 (2013). 23032747 10.1158/1078-0432.CCR-12-1139

[38] Zhuang, X. et al. Ageing limits stemness and tumorigenesis by reprogramming iron homeostasis. *Nature* **637**, 184–194 (2025). 39633048 10.1038/s41586-024-08285-0

[39] Shuldiner, E. G. et al. Aging represses oncogenic KRAS-driven lung tumorigenesis and alters tumor suppression. *Nat. Aging* **5**, 2263–2278 (2025). 41188600 10.1038/s43587-025-00986-z PMC12616358

[40] Bray, F. et al. Global cancer statistics 2022: GLOBOCAN estimates of incidence and mortality worldwide for 36 cancers in 185 countries. *CA Cancer J. Clin.* **74**, 229–263 (2024). 38572751 10.3322/caac.21834

[41] Tesfaw, L. M., Dessie, Z. G. & Mekonnen Fenta, H. Lung cancer mortality and associated predictors: systematic review using 32 scientific research findings. *Front. Oncol.* **13**, 1308897 (2023). 38156114 10.3389/fonc.2023.1308897 PMC10754488

[42] Eyles, J. et al. Tumor cells disseminate early, but immunosurveillance limits metastatic outgrowth, in a mouse model of melanoma. *J. Clin. Invest.* **120**, 2030–2039 (2010). 20501944 10.1172/JCI42002 PMC2877955

[43] McCreery, M. Q. et al. Evolution of metastasis revealed by mutational landscapes of chemically induced skin cancers. *Nat. Med.* **21**, 1514–1520 (2015). 26523969 10.1038/nm.3979 PMC5094808

[44] Rhim, A. D. et al. EMT and dissemination precede pancreatic tumor formation. *Cell* **148**, 349–361 (2012). 22265420 10.1016/j.cell.2011.11.025 PMC3266542

[45] Hosseini, H. et al. Early dissemination seeds metastasis in breast cancer. *Nature* **540**, 552–558 (2016). 27974799 10.1038/nature20785 PMC5390864

[46] Werner-Klein, M. et al. Genetic alterations driving metastatic colony formation are acquired outside of the primary tumour in melanoma. *Nat. Commun.* **9**, 595 (2018). 29426936 10.1038/s41467-017-02674-y PMC5807512

[47] Al Bakir, M. et al. The evolution of non-small cell lung cancer metastases in TRACERx. *Nature* **616**, 534–542 (2023). 37046095 10.1038/s41586-023-05729-x PMC10115651

[48] Schneider, J. L. et al. The aging lung: physiology, disease, and immunity. *Cell* **184**, 1990–2019 (2021). 33811810 10.1016/j.cell.2021.03.005 PMC8052295

[49] Sharma, G. & Goodwin, J. Effect of aging on respiratory system physiology and immunology. *Clin. Interv. Aging* **1**, 253–260 (2006). 18046878 10.2147/ciia.2006.1.3.253 PMC2695176

[50] Rowbotham, S. P. et al. Age-associated H3K9me2 loss alters the regenerative equilibrium between murine lung alveolar and bronchiolar progenitors. *Dev. Cell* **58**, 2974–2991 (2023). 37977149 10.1016/j.devcel.2023.10.011 PMC10873032

[51] Angelidis, I. et al. An atlas of the aging lung mapped by single cell transcriptomics and deep tissue proteomics. *Nat. Commun.* **10**, 963 (2019). 30814501 10.1038/s41467-019-08831-9 PMC6393476

[52] Wiel, C. et al. BACH1 stabilization by antioxidants stimulates lung cancer metastasis. *Cell* **178**, 330–345 (2019). 31257027 10.1016/j.cell.2019.06.005

[53] Bergers, G. & Fendt, S. M. The metabolism of cancer cells during metastasis. *Nat. Rev. Cancer* **21**, 162–180 (2021). 33462499 10.1038/s41568-020-00320-2 PMC8733955

[54] Tasdogan, A. et al. Metabolic heterogeneity confers differences in melanoma metastatic potential. *Nature* **577**, 115–120 (2020). 31853067 10.1038/s41586-019-1847-2 PMC6930341

[55] Ward, C. P. et al. Aging alters the metabolic flux signature of the ER-unfolded protein response in vivo in mice. *Aging Cell* **21**, e13558 (2022). 35170180 10.1111/acel.13558 PMC8920450

[56] Jin, J., Byun, J. K., Choi, Y. K. & Park, K. G. Targeting glutamine metabolism as a therapeutic strategy for cancer. *Exp. Mol. Med.* **55**, 706–715 (2023). 37009798 10.1038/s12276-023-00971-9 PMC10167356

[57] Moqri, M. et al. Validation of biomarkers of aging. *Nat. Med.* **30**, 360–372 (2024). 38355974 10.1038/s41591-023-02784-9 PMC11090477

[58] Hill, W., Caswell, D. R. & Swanton, C. Capturing cancer evolution using genetically engineered mouse models (GEMMs). *Trends Cell Biol.* **31**, 1007–1018 (2021). 34400045 10.1016/j.tcb.2021.07.003

[59] Zhang, F. et al. Modification of the telomerase gene with human regulatory sequences resets mouse telomeres to human length. *Nat. Commun.* **16**, 1211 (2025). 39905075 10.1038/s41467-025-56559-6 PMC11794480

[60] Jackson, E. L. et al. Analysis of lung tumor initiation and progression using conditional expression of oncogenic K-ras. *Genes Dev.* **15**, 3243–3248 (2001). 11751630 10.1101/gad.943001 PMC312845

[61] Romero, R. et al. Keap1 loss promotes Kras-driven lung cancer and results in dependence on glutaminolysis. *Nat. Med.* **23**, 1362–1368 (2017). 28967920 10.1038/nm.4407 PMC5677540

[62] Geissmann, Q. OpenCFU a new free and open-source software to count cell colonies and other circular objects. *PLoS ONE* **8**, e54072 (2013). 10.1371/journal.pone.0054072 PMC3574151 23457446

[63] Shalem, O. et al. Genome-scale CRISPR-Cas9 knockout screening in human cells. *Science* **343**, 84–87 (2014). 24336571 10.1126/science.1247005 PMC4089965

[64] Sanjana, N. E., Shalem, O. & Zhang, F. Improved vectors and genome-wide libraries for CRISPR screening. *Nat. Methods* **11**, 783–784 (2014). 25075903 10.1038/nmeth.3047 PMC4486245

[65] Joung, J. et al. A transcription factor atlas of directed differentiation. *Cell* **186**, 209–229 (2023). 36608654 10.1016/j.cell.2022.11.026 PMC10344468

[66] Fellmann, C. et al. An optimized microRNA backbone for effective single-copy RNAi. *Cell Rep.* **5**, 1704–1713 (2013). 24332856 10.1016/j.celrep.2013.11.020

[67] Lewis, C. A. et al. Tracing compartmentalized NADPH metabolism in the cytosol and mitochondria of mammalian cells. *Mol. Cell* **55**, 253–263 (2014). 10.1016/j.molcel.2014.05.008 PMC4106038 24882210

[68] Fernandez, C. A., Des Rosiers, C., Previs, S. F., David, F. & Brunengraber, H. Correction of 13 C mass isotopomer distributions for natural stable isotope abundance. *J. Mass Spectrom.* **31**, 255–262 (1996). 8799277 10.1002/(SICI)1096-9888(199603)31:3<255::AID-JMS290>3.0.CO;2-3

[69] Picelli, S. et al. Smart-seq2 for sensitive full-length transcriptome profiling in single cells. *Nat. Methods* **10**, 1096–1098 (2013). 24056875 10.1038/nmeth.2639

[70] Landberg, G. et al. Characterization of cell-free breast cancer patient-derived scaffolds using liquid chromatography-mass spectrometry/mass spectrometry data and RNA sequencing data. *Data Brief* **31**, 105860 (2020). 32637480 10.1016/j.dib.2020.105860 PMC7327418

[71] Dobin, A. et al. STAR: ultrafast universal RNA-seq aligner. *Bioinformatics* **29**, 15–21 (2013). 23104886 10.1093/bioinformatics/bts635 PMC3530905

[72] Anders, S., Pyl, P. T. & Huber, W. HTSeq—a Python framework to work with high-throughput sequencing data. *Bioinformatics* **31**, 166–169 (2015). 25260700 10.1093/bioinformatics/btu638 PMC4287950

[73] Love, M. I., Huber, W. & Anders, S. Moderated estimation of fold change and dispersion for RNA-seq data with DESeq2. *Genome Biol.* **15**, 550 (2014). 25516281 10.1186/s13059-014-0550-8 PMC4302049

[74] Subramanian, A. et al. Gene set enrichment analysis: a knowledge-based approach for interpreting genome-wide expression profiles. *Proc. Natl Acad. Sci. USA* **102**, 15545–15550 (2005). 16199517 10.1073/pnas.0506580102 PMC1239896

[75] Sherman, B. T. et al. DAVID: a web server for functional enrichment analysis and functional annotation of gene lists (2021 update). *Nucleic Acids Res.* **50**, W216–w221 (2022). 35325185 10.1093/nar/gkac194 PMC9252805

[76] Sápi, J. et al. Tumor volume estimation and quasi-continuous administration for most effective bevacizumab therapy. *PLoS ONE* **10**, e0142190 (2015). 26540189 10.1371/journal.pone.0142190 PMC4635016

[77] Broad DepMap. DepMap 23Q2 Public. *figshare*10.6084/m9.figshare.22765112 (2023).

[78] Flurkey, K. et al. in *The Mouse in Biomedical Research* 2nd edn (eds Fox, J. G. et al.) 637–672 (Academic, 2007).

