# Supplementary material for: Ageing promotes metastasis via activation of the integrated stress response
Source: Nature. 2026 Mar 11;652(8112):1339–48. doi: 10.1038/s41586-026-10216-0 (PMC13128440; doi:10.1038/s41586-026-10216-0)

nature portfolio

https://doi.org/10.1038/s41586-026-10216-0

Supplementary information

Ageing promotes metastasis via activation of the integrated stress response

In the format provided by the authors and unedited

Uncropped Western Blot; Patel et. al

Figure 1f

ACTIN as sample processing control

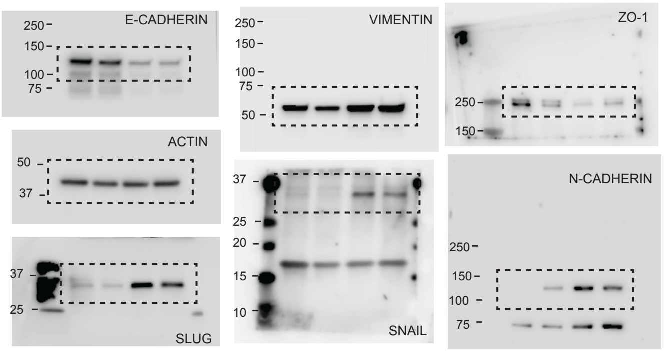

Figure 2d

HISTONE3 and HSP90 as loading controls

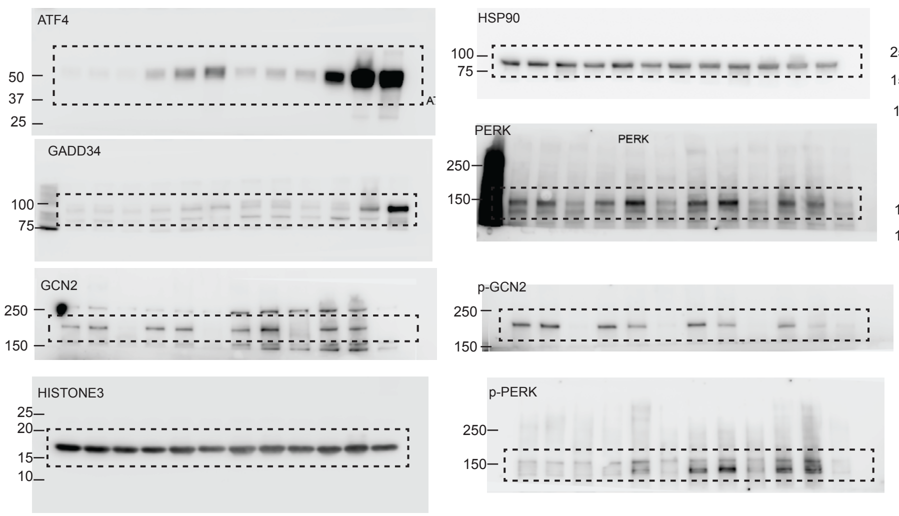

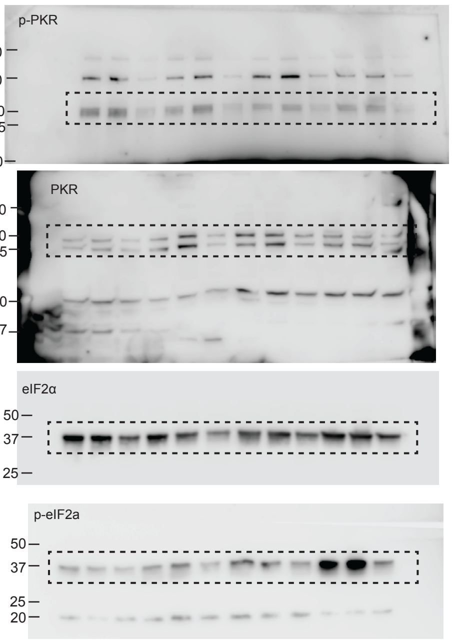

Figure 2f

HISTONE3 as loading control

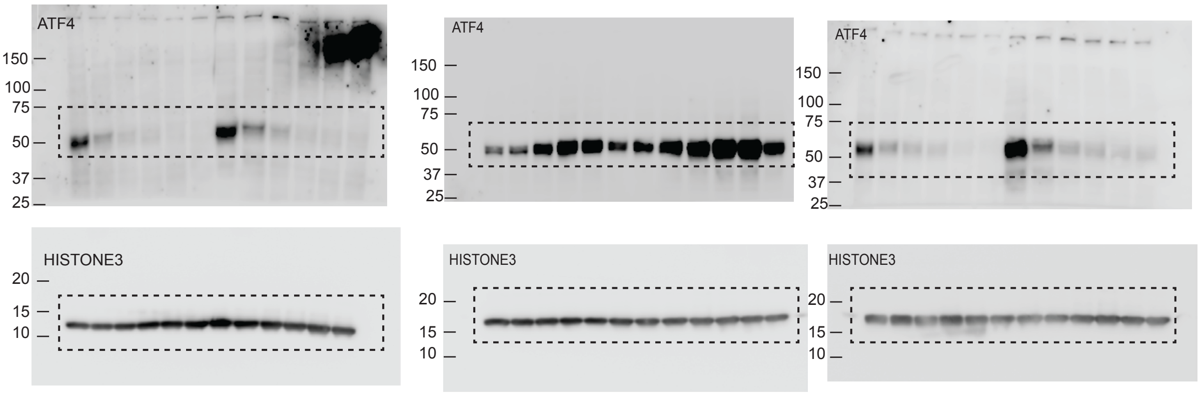

Figure 3a

HISTONE3 as loading control

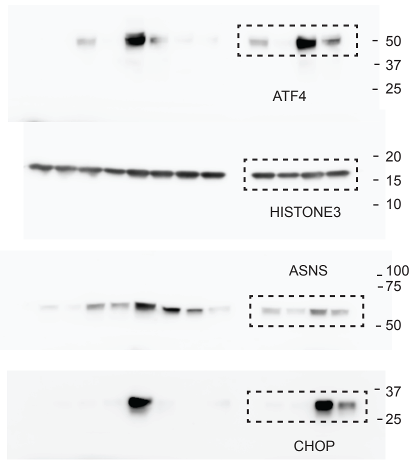

Figure 3d

HSP90 as loading control

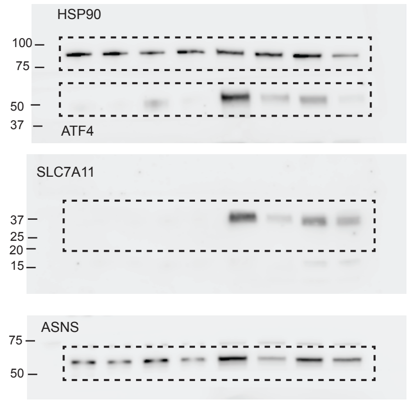

Figure 3j

HISTONE3 as loading control

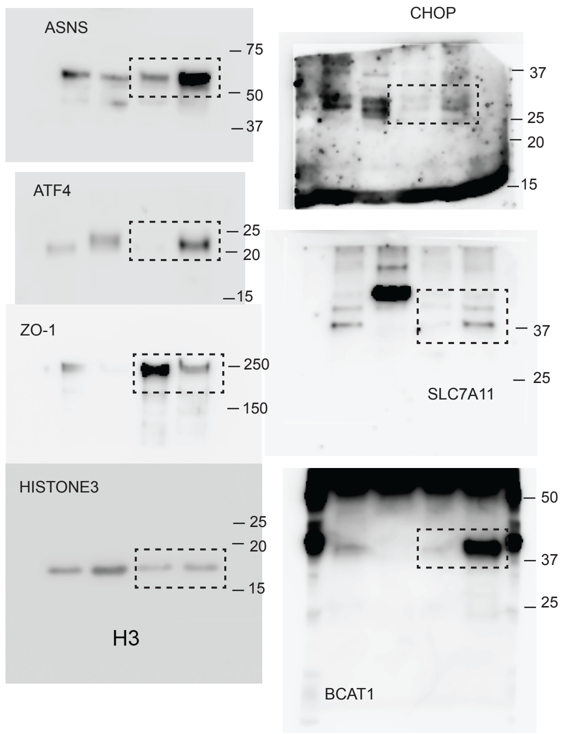

Figure 3g

ACTIN as sample processing control

HISTONE3 as loading control

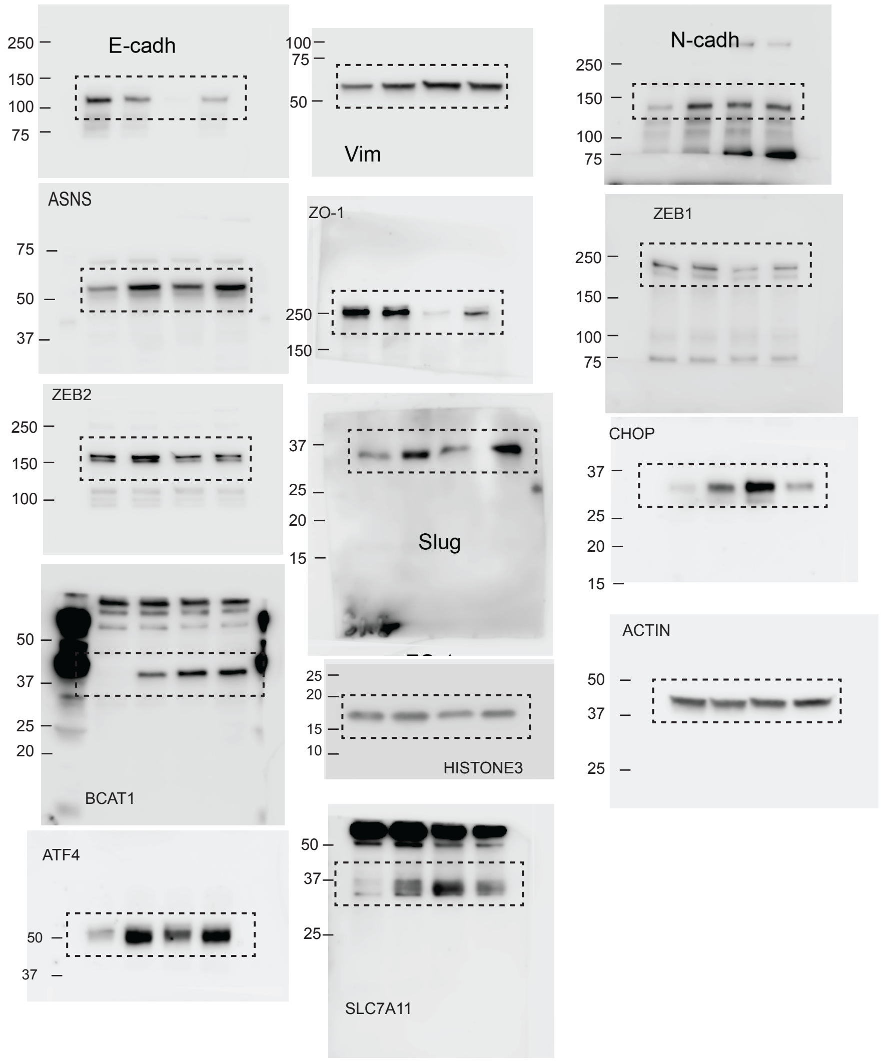

Extended Data Figure 2d

HISTONE3 as sample processing control

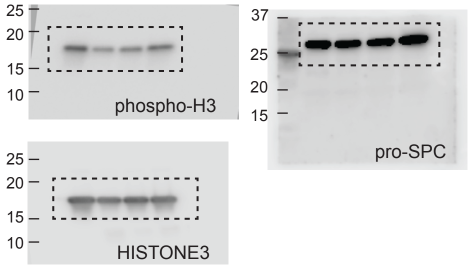

Extended Data Figure 3d

ACTIN as loading control

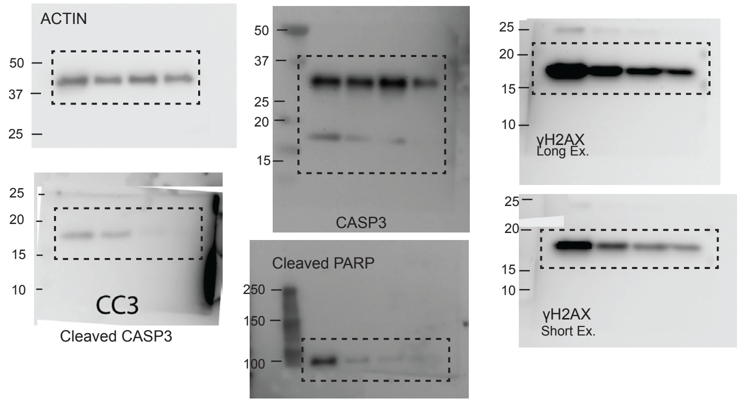

Extended Data Figure 5i

HISTONE3 as loading control

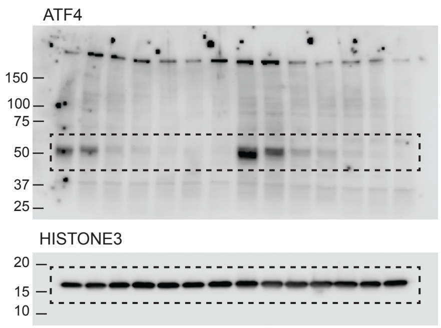

Extended Data Figure 5k

HSP90 as loading control

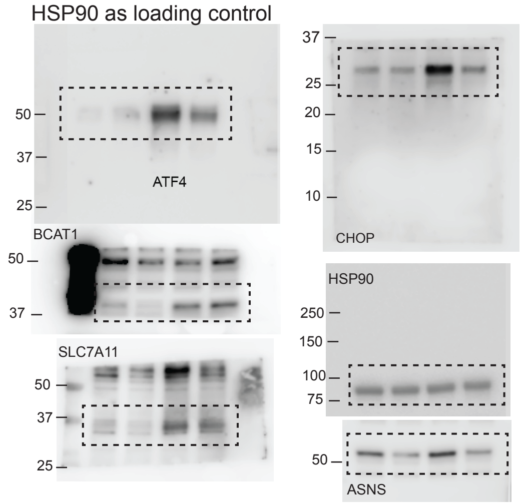

Extended Data Figure 5j

ACTIN and HSP90 as loading controls

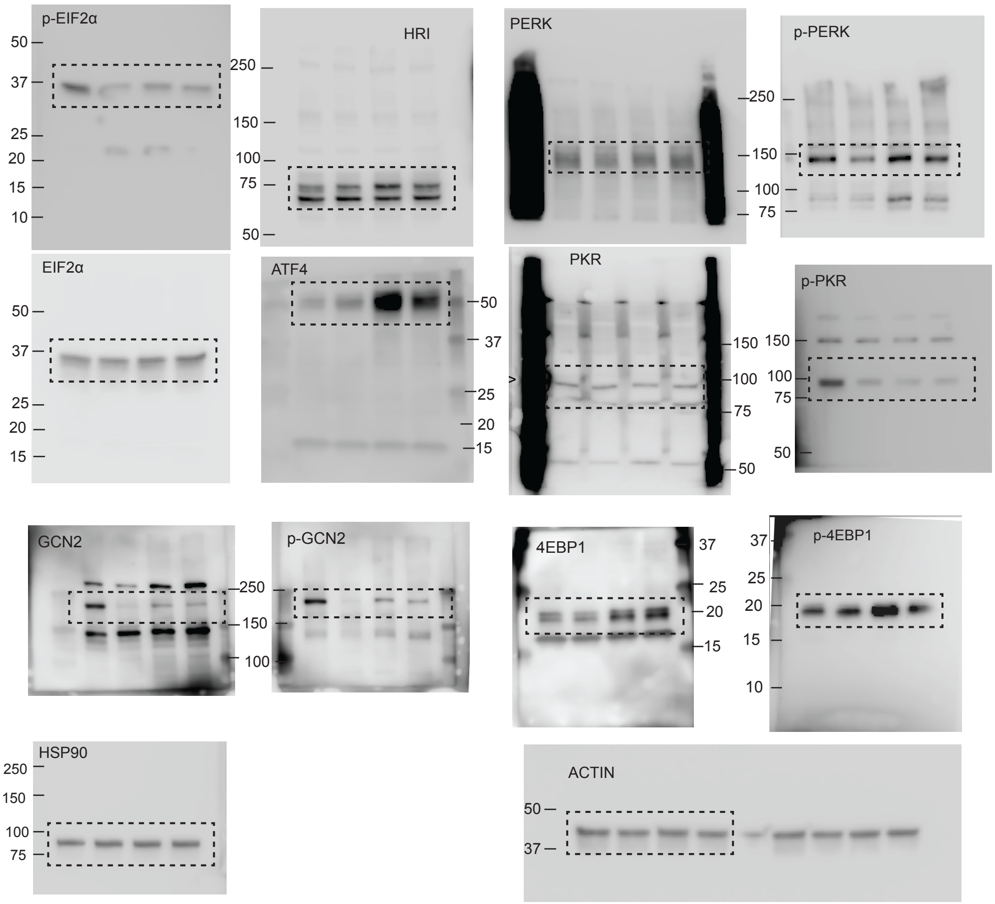

Extended Data Figure 6k

HSP90 as loading control

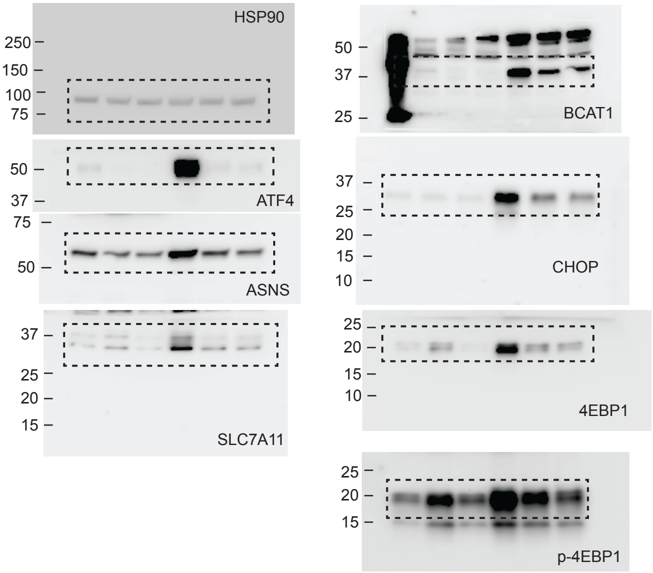

Extended Data Figure 6r

TUBULIN as loading control

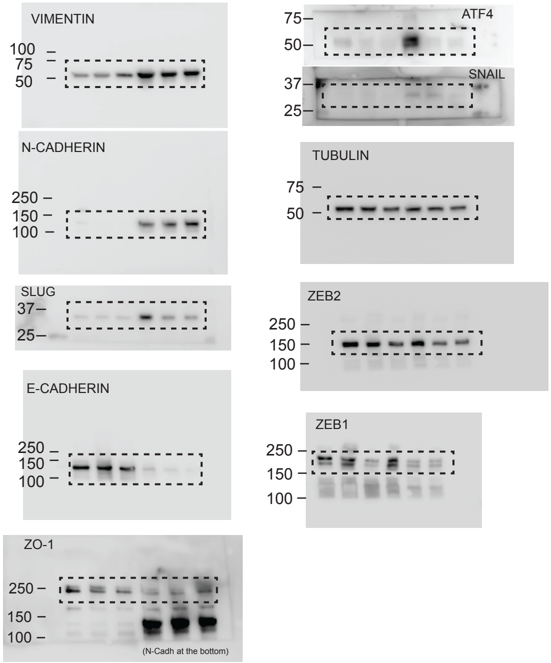

Supplement: Supplementary file 1 — Gel source data. All uncropped western blot images with protein ladder markers. [file 41586_2026_10216_MOESM1_ESM.pdf]
