# Supplementary material for: Ageing promotes metastasis via activation of the integrated stress response
Source: Nature. 2026 Mar 11;652(8112):1339–48. doi: 10.1038/s41586-026-10216-0 (PMC13128440; doi:10.1038/s41586-026-10216-0)
Supplement: Supplementary file 2 — Reporting Summary [file 41586_2026_10216_MOESM2_ESM.pdf]

Corresponding author(s): DAPR ms 2024-06-12120B

Last updated by author(s): Jan 23, 2026

Reporting Summary

Nature Portfolio wishes to improve the reproducibility of the work that we publish. This form provides structure for consistency and transparency in reporting. For further information on Nature Portfolio policies, see our Editorial Policies and the Editorial Policy Checklist.

Statistics

For all statistical analyses, confirm that the following items are present in the figure legend, table legend, main text, or Methods section.

n/a	Confirmed	
☐	☒	The exact sample size (n) for each experimental group/condition, given as a discrete number and unit of measurement
☒	☐	A statement on whether measurements were taken from distinct samples or whether the same sample was measured repeatedly
☐	☒	The statistical test(s) used AND whether they are one- or two-sided<br>*Only common tests should be described solely by name; describe more complex techniques in the Methods section.*
☒	☐	A description of all covariates tested
☐	☒	A description of any assumptions or corrections, such as tests of normality and adjustment for multiple comparisons
☐	☒	A full description of the statistical parameters including central tendency (e.g. means) or other basic estimates (e.g. regression coefficient) AND variation (e.g. standard deviation) or associated estimates of uncertainty (e.g. confidence intervals)
☐	☒	For null hypothesis testing, the test statistic (e.g. F, t, r) with confidence intervals, effect sizes, degrees of freedom and P value noted<br>*Give P values as exact values whenever suitable.*
☒	☐	For Bayesian analysis, information on the choice of priors and Markov chain Monte Carlo settings
☒	☐	For hierarchical and complex designs, identification of the appropriate level for tests and full reporting of outcomes
☐	☒	Estimates of effect sizes (e.g. Cohen's d, Pearson's r), indicating how they were calculated

Our web collection on statistics for biologists contains articles on many of the points above.

Software and code

Policy information about availability of computer code

Data collection	Histological, Immunohistochemistry images were acquired using Olympus Slideview VS200 and Hamamatzu Nanozoomer (2.0HT). 2D cell culture and 3D spheroid images were acquired using Zeiss Axio Observer.A1 reverse microscope with an AxioCam MRm camera (Carl Zeiss). Flow cytometry data was acquired using Sony ID7000™ Spectral Cell Analyzer. Western Blot imaging was carried out Amersham ImageQuant 800 Western blot imaging systems (Cytiva).Quantitative RT-PCR data was collected using QuantStudio™ 5 Real-Time PCR system (2.5.1). Oxygen Consumption Rate (OCR) and Extracellular Acidification Rate (ECAR) was acquired using XF96pro apparatus from Seahorse Bioscience (Agilent). For Metabolomics tracing, Agilent Intuvo gas chromatograph coupled to an Agilent 5977B mass spectrometer (Agilent Technologies) was used. RNA sequencing data was performed with NextSeq 500 instrument (Illumina). ATAC-sequencing was performed with HiSeq (Illumina).Data from IVIS imaging was collected using Perkin Elmer Living Image software (4.7.4). Data from cell viability assays were collected using Synegry BioTex HTX multi-mode spectrophotometer.

Data analysis

Statistical Analysis was done and plotted using GraphPad Prism (v 9.0 and v 10.0).
Flow cytometry analysis was done using Flow Jo software (10.8.1).
Hematoxylin and Eosin (H&E) sections were analysed using BioPix iQ software (v 2.1.4)
Seahorse data was analysed using Wave Desktop (v2.6).
For RNA-seq Alignment of sequencing reads was performed using HTSeq version 0.9.1, STAR version 2.7.9a, R package (v 4.1.2), DESeq2 (v 1.34.0), Geneset Enrichment Analysis (GSEA v 4.3.3).
For ATAC-seq, Trimmomatic (v 0.38), Bowtie2 (v 2.5.2), samtools (1.9), Picard (2.18.26), MACS2 (2.1.2), Diffbind (v 3.20.0), Integrated Genomic Viewer (IGV 2.16.2) and web-based Genomic Regions of Enrichment of Annotations Tool (GREAT).
IVIS imaging data was analysed using Perkin Elmer Living Image software (4.7.4).
Adobe Illustrator (v 30.1) was used for schematics.

For manuscripts utilizing custom algorithms or software that are central to the research but not yet described in published literature, software must be made available to editors and reviewers. We strongly encourage code deposition in a community repository (e.g. GitHub). See the Nature Portfolio guidelines for submitting code & software for further information.

Data

Policy information about availability of data

All manuscripts must include a data availability statement. This statement should provide the following information, where applicable:

- Accession codes, unique identifiers, or web links for publicly available datasets
- A description of any restrictions on data availability
- For clinical datasets or third party data, please ensure that the statement adheres to our policy

RNASeq raw and associated processed data from primary KP-Y and KP-O cultures with or without ATF4 over-expression and RNA-seq, ATAC-seq raw and associated processed data from KP-Y and KP-O primary cultures have been deposited in the Gene Expression Omnibus (GEO) under accession codes GSE263689 (will need to add link). All the other data and raw gel images are included with the paper. All the databases used in this study are publicly available. Gene expression profiles and clinical information for lung adenocarcinoma patients were obtained from The Cancer Genome Atlas lung adenocarcinoma cohort (TCGA-LUAD; https://www.cancer.gov/tcga). Genomic alteration and clinical data were accessed through the cBioPortal for Cancer Genomics (https://www.cbioportal.org). Information regarding lung adenocarcinoma organotropism was obtained from MSK-IMPACT data 41. Gene expression and dependency data for cancer cell lines were obtained from the Cancer Cell Line Encyclopedia (CCLE) and DepMap portal (version 1, Public 23Q2; https://depmap.org/portal.84 Population doubling times and cell line age annotations were retrieved from the Cellosaurus database (https://www.cellosaurus.org/). Protein–protein interaction analyses were performed using the STRING database (https://string-db.org/). Source data are provided with this paper.

Research involving human participants, their data, or biological material

Policy information about studies with human participants or human data. See also policy information about sex, gender (identity/presentation), and sexual orientation and race, ethnicity and racism.

Reporting on sex and gender	All available patient characteristics is provided are Extended Data Table 1 and Table 2.
Reporting on race, ethnicity, or other socially relevant groupings	N/A
Population characteristics	All available patient characteristics are provided in Extended Data Table 1 and Table 2.
Recruitment	For retrospective data all patients in West Sweden (Västra Götalandsregionen) diagnosed with NSCLC from the 2016 to 2018; Tissue microarrays (TMAs) were obtained from the Swedish NSCLC cohort included primary NSCLC resections (1995–2005).
Ethics oversight	Ethical approval was obtained from the Swedish Ethical Review Authority (DNR 2019-04711 and 2021-04987).<br>TMAs collection from NSCLC resected patients complied with Swedish legislation and were approved by the Uppsala Ethical Review Board (2006/325). No informed consent was required due to all data are in a de-identified form, according to the Swedish Ethical Review Authority.

Note that full information on the approval of the study protocol must also be provided in the manuscript.

Field-specific reporting

Please select the one below that is the best fit for your research. If you are not sure, read the appropriate sections before making your selection.

☒ Life sciences ☐ Behavioural & social sciences ☐ Ecological, evolutionary & environmental sciences

For a reference copy of the document with all sections, see nature.com/documents/nr-reporting-summary-flat.pdf

Life sciences study design

All studies must disclose on these points even when the disclosure is negative.

Sample size

At least n = 3 biological replicates were used per condition for each in vitro experiment. No formal power analysis was performed for in vitro

Sample size	experiments. For in vivo experiments, a minimum of n = 3 animals was used per group for each experiment. Exact sample sizes are reported in the corresponding figure legends. Sample sizes were chosen based on prior experience and previous experiments demonstrating the ability to detect statistically significant differences between conditions with groups balanced for sex whenever possible.
Data exclusions	For Fig. 4p-q, 3 mice from the KP-O group were excluded from the study due to mortality before the endpoint, caused by factors unrelated to tumor and metastasis formation.
Replication	All in vitro experiments were reproduced at least three times and whenever possible automated quantifications were performed using the appropriate software and the attempts were successful. Western blot experiments were replicated at least three times with reproducible results. When representative images are shown, a minimum of three samples per group from the larger cohort were evaluated.
Randomization	Samples and experimental mice were randomly assigned to experimental groups. Sample collection was also assigned randomly
Blinding	The investigators were not blinded for the allocation of the groups during experiments or during in vitro/in vivo experiments, as such knowledge is essential to conduct experiments.

Reporting for specific materials, systems and methods

We require information from authors about some types of materials, experimental systems and methods used in many studies. Here, indicate whether each material, system or method listed is relevant to your study. If you are not sure if a list item applies to your research, read the appropriate section before selecting a response.

Materials & experimental systems

n/a	Involved in the study	
☐	☒	Antibodies
☐	☒	Eukaryotic cell lines
☒	☐	Palaeontology and archaeology
☐	☒	Animals and other organisms
☐	☒	Clinical data
☒	☐	Dual use research of concern
☐	☐	Plants

Methods

n/a	Involved in the study	
☒	☐	ChIP-seq
☒	☐	Flow cytometry
☒	☐	MRI-based neuroimaging

Antibodies

Antibodies used

All antibodies are described below. All antibodies were purchased from Cell Signaling, Abcam, Proteintech, Jackson Immunoreserach, Sigma Aldrich. All antibodies were validated by manufacturers and in previous publications.
Antibodies for Western Blot
Actin (1:5000) Sigma Aldrich A2228
HSP90 (1:1000) Cell Signaling 4874
SLC7A11 (xCT) (1:1000) Cell Signaling 98051S
Histone H3 (1:20000) Abcam Ab1791
Phospho-HISTONE H3 (1:1000) Cell signaling 9718
CASPASE 3 (1:500) Cell signaling 9662
Cleaved CASPASE 3 (1:500) Cell Signaling 9664
Cleaved PARP (1:1000) Cell Signaling 9548
VIMENTIN (D21H3) (1:1000) Cell Signaling 5741
E-CADHERIN (24E10) (1:1000) Cell Signaling 3195
N-CADHERIN (1:1000) Cell Signaling 13116
SNAIL (1:1000) Cell Signaling 3879
SLUG (1:1000) Cell Signaling 9585
ZO-1 (1:1000) Cell Signaling 8193
EiF2a (1:1000) Cell Signaling 5324
Phospho-EiF2a (1:1000) Abcam AB32157
4E-BP1 (1:1000) Cell Signaling 9452
Phospho-4E-BP1 (1:1000) Cell Signaling 9451
ATF4 (D4B8) (1:1000) Cell Signaling 11815S
PSAT1 (1:1000) Proteintech 20180-1-AP-150
ASNS (1:1000) Proteintech 14681-1-AP-150
BCAT1 (1:1000) Proteintech 13640-1-AP-150
CHOP (1:500) Proteintech 15204-1-AP-20
γH2AX (1:500) Cell Signaling 9718
phospho-GCN2 (1:1000) Cell Signaling 94668
GCN2 (1:1000) Cell Signaling 3302
HRI (1:1000) Proteintech 20499-1-AP-20
phospho-PKR (1:1000) Abcam AB32036
PKR (1:1000) Proteintech 18244-1-AP-20
phospho-PERK (1:1000) Proteintech 29546-1-AP-20
PERK (1:1000) Proteintech 24390-1-AP-20
GADD34 (1:5000) Proteintech 10449-1-AP

ZEB1 (1:1000) Cell Signaling 3396P
ZEB2 (1:1000) Cell Signaling 97885
TUBULIN (1:5000) Sigma-Aldrich T6199
HRP-conjugated anti-Rabbit (1:10000) Jackson Immunoresearch 111-035-003
HRP-conjugated anti-Mouse Jackson (1:10000) Immunoresearch 115-035-003
Antibodies for ImmunohistoChemistry
pro-SPC (1:500) Sigma-Aldrich AB3786
ASNS (1:100) Proteintech 14681-1-AP-150
SLC7A11 (1:100) Proteintech 26864-1-AP-150
Phospho-HISTONE H3 (1:200) Cell Signaling 9718
ATF4 (1:50) Cell Signaling 118154S
ATF4 (1:200) Invitrogen MA5-33117
HMGA2 (1:500) Proteintech 20795-1-AP
Anti-TTF1/Nkx2-1 antibody [EP1584Y] (1:500) abcam ab76013

Validation

Actin;https://www.sigmaaldrich.com/SE/en/product/sigma/a2228
HSP90; https://www.cellsignal.com/products/primary-antibodies/hsp90-antibody/4874
Slc7a11;https://www.cellsignal.com/products/primary-antibodies/xct-slc7a11-antibody/98051
Histone H3;https://www.abcam.com/en-se/products/primary-antibodies/histone-h3-antibody-nuclear-marker-and-chip-grade-ab1791
Phospho-HISTONE 3;https://www.cellsignal.com/products/primary-antibodies/phospho-histone-h2a-x-ser139-20e3-rabbit-mab/9718
CASPASE 3;https://www.cellsignal.com/products/primary-antibodies/caspase-3-antibody/9662
Cleaved CASPASE 3;https://www.cellsignal.com/products/primary-antibodies/cleaved-caspase-3-asp175-5a1e-rabbit-mab/9664
Cleaved PARP;https://www.cellsignal.com/products/primary-antibodies/cleaved-parp-asp214-7c9-mouse-mab/9548
VIMENTIN (D21H3);https://www.cellsignal.com/products/primary-antibodies/vimentin-d21h3-xp-rabbit-mab/5741
E-CADHERIN (24E10);https://www.cellsignal.com/products/primary-antibodies/e-cadherin-24e10-rabbit-mab/3195
N-CADHERIN;https://www.cellsignal.com/products/primary-antibodies/n-cadherin-d4r1h-xp-rabbit-mab/13116
SNAIL;https://www.cellsignal.com/products/primary-antibodies/snail-c15d3-rabbit-mab/3879
SLUG; https://www.cellsignal.com/products/primary-antibodies/slug-c19g7-rabbit-mab/9585
ZO-1; https://www.cellsignal.com/products/primary-antibodies/zo-1-d7d12-rabbit-mab/8193
EiF2a;https://www.cellsignal.com/products/primary-antibodies/eif2a-d7d3-xp-rabbit-mab/5324
Phospho-EiF2a;https://www.abcam.com/en-se/products/primary-antibodies/eif2s1-phospho-s51-antibody-e90-ab32157
4E-BP1;https://www.cellsignal.com/products/primary-antibodies/4e-bp1-antibody/9452
Phospho-4E-BP1;https://www.cellsignal.com/products/primary-antibodies/phospho-4e-bp1-ser65-antibody/9451
ATF4 (D4B8);https://www.cellsignal.com/products/primary-antibodies/atf-4-d4b8-rabbit-mab/11815
PSAT1;https://www.ptglab.com/products/PSAT1-Antibody-20180-1-AP.htm
ASNS;https://www.ptglab.com/products/ASNS-Antibody-14681-1-AP.htm
BCAT1;https://www.ptglab.com/products/BCAT1-Antibody-13640-1-AP.htm
CHOP;https://www.ptglab.com/products/DDIT3-Antibody-15204-1-AP.htm
γH2AX;https://www.cellsignal.com/products/primary-antibodies/phospho-histone-h2a-x-ser139-20e3-rabbit-mab/9718
phospho-GCN2;https://www.cellsignal.com/products/primary-antibodies/phospho-gcn2-thr899-e1v9m-rabbit-mab/94668
GCN2;https://www.cellsignal.com/products/primary-antibodies/gcn2-antibody/3302
HRI;https://www.ptglab.com/products/EIF2AK1-Antibody-20499-1-AP.htm
phospho-PKR;https://www.abcam.com/en-se/products/primary-antibodies/pkr-phospho-t446-antibody-e120-ab32036
PKR;https://www.ptglab.com/products/Phospho-PERK-EIF2AK3-Ser719-Antibody-29546-1-AP.htm
phospho-PERK;https://www.ptglab.com/products/Phospho-PERK-EIF2AK3-Ser719-Antibody-29546-1-AP.htm
PERK;https://www.ptglab.com/products/EIF2AK3-Antibody-24390-1-AP.htm
GADD34; https://www.ptglab.com/products/PPP1R15A,GADD34-Antibody-10449-1-AP
ZEB1; https://www.cellsignal.com/products/primary-antibodies/zeb1-d80d3-rabbit-mab/3396
ZEB2; https://www.cellsignal.com/products/primary-antibodies/zeb2-e6u7z-rabbit-mab/97885
TUBULIN; https://www.sigmaaldrich.com/SE/en/product/sigma/t6199
HRP-conjugated anti-Rabbit ;https://www.jacksonimmuno.com/catalog/products/111-035-003
HRP-conjugated anti-Mouse;https://www.jacksonimmuno.com/catalog/products/115-035-003

For Immunohistochemistry
pro-SPC;https://www.sigmaaldrich.com/SE/en/product/mm/ab3786
ASNS;https://www.ptglab.com/products/ASNS-Antibody-14681-1-AP.htm
SLC7A11;https://www.ptglab.com/products/xCT-Antibody-26864-1-AP.htm
Phospho-HISTONE 3;https://www.cellsignal.com/products/primary-antibodies/phospho-histone-h2a-x-ser139-20e3-rabbit-mab/9718
ATF4;https://www.cellsignal.com/products/primary-antibodies/atf-4-d4b8-rabbit-mab/11815.
ATF4; https://www.thermofisher.com/antibody/product/ATF4-Antibody-clone-9E1-Recombinant-Monoclonal/MA5-33117
HMGA2; https://www.ptglab.com/products/HMGA2-Antibody-20795-1-AP.htm
Anti-TTF1/Nkx2-1; https://www.abcam.com/en-us/products/primary-antibodies/ttf1-nkx2-1-antibody-ep1584y-ab76013.

Eukaryotic cell lines

Policy information about cell lines and Sex and Gender in Research

Cell line source(s)

Primary cultures were isolated from lung tumors of KP-Young and KP-Old mice; HEK293FT cells were purchased from Life Technologies (#R70007); A549 cells, human lung adenocarcinoma epithelial cell line, was obtained from the American Type Culture Collection (ATCC, catalog no. CCL-185)

Authentication

Primary cultures were not authenticated. A549 and HEK293FT cell lines was frequently checked by its morphological features.

Mycoplasma contamination	Cells were routinely tested for mycoplasma and tested negative. Testing was done on a weekly basis.
Commonly misidentified lines (See ICLAC register)	No commonly misidentified cell lines were used.

Animals and other research organisms

Policy information about studies involving animals; ARRIVE guidelines recommended for reporting animal research, and Sex and Gender in Research

Laboratory animals	KrasLSL-G12D/+ Trp53flox/flox (KP) mice were mixed C57BL/6-129/Sv genetic background from Jackson Lab (Strain #:032435; RRID:IMSR_JAX:032435); Young KP (3 months old) while old KP (> 16 months old). NXG mice (NOD-Prkdcscid -IL2rgTm1 381 / Rj) were obtained from Janvier Labs and were 6-10 weeks old at the start of the experiments. Same-sex mice were housed in individually ventilated cages (IVC), under a 12 h–12 h light-dark cycle, with ambient temperature (15 - 21℃) and humidity control (45-70% RH), enrichment material, and ab libitum rodent chow and water.
Wild animals	No wild animals were used.
Reporting on sex	No findings applied only to one sex, and similar number of male and female mice have been used.
Field-collected samples	No field collected samples were used in the study.
Ethics oversight	All mouse experiments described in this study were approved by the Research Animal Ethics Committee in Gothenburg (2071/19; 2077/19 and 6057/24).

Note that full information on the approval of the study protocol must also be provided in the manuscript.

Clinical data

Policy information about clinical studies

All manuscripts should comply with the ICMJE guidelines for publication of clinical research and a completed CONSORT checklist must be included with all submissions.

Clinical trial registration	N/A
Study protocol	N/A
Data collection	All patients in West Sweden (Västra Götalandsregionen) diagnosed with NSCLC from the years 2016 to 2018 and molecularly assessed were included (n = 997). Age at diagnosis, histology, and tumor size at diagnosis from CT scans were obtained from patient charts. Patient demographics (age, sex, Eastern Cooperative Oncology Group [ECOG] performance status [PS], and smoking history), cancer stage, pathological details (histology, mutational status including KRAS mutational status and subtype) were retrospectively collected from patient charts and the Swedish Lung Cancer Registry. Clinical staging was based on TNM staging guidelines 7th edition. For TMAs, KRAS-mutant lung adenocarcinomas were selected; ATF4 expression was scored and correlation with survival outcomes analyzed.
Outcomes	N/A

Plants

Seed stocks	N/A
Novel plant genotypes	N/A
Authentication	N/A
